# Comparison Between Binuclear and Mononuclear Ru(II) Complexes: Synthesis, Structure, Photophysics, and Oxygen Sensing Performance

**DOI:** 10.3389/fchem.2021.785309

**Published:** 2021-11-24

**Authors:** Fei Wang, Liyuan Yang, Xue-Quan Xian

**Affiliations:** ^1^ School of Mechanical and Electrical Engineering, Zhengzhou University of Industrial Technology, Zhengzhou, China; ^2^ School of Material Science and Engineering, Zhengzhou University, Zhengzhou, China; ^3^ National Engineering Research Center for Non-Food Biorefinery, State Key Laboratory of Non-Food Biomass and Enzyme Technology, Guangxi Key Laboratory of Biorefinery, Guangxi Academy of Sciences, Nanning, China

**Keywords:** multinuclear Ru(II), mononuclear Ru(II), linear response, optical oxygen sensing, electrospun fiber

## Abstract

Owing to their attractive potential in optoelectronic application, luminescent Ru(II) complexes with diamine ligands are harvesting more and more research efforts. These literature efforts, however, are mostly mononuclear ones, with no detailed discussion on the performance comparison between mononuclear and multinuclear Ru(II) complexes. This work synthesized three diamine ligands having two or multiple chelating sites in each ligand, as well as their Ru(II) complexes. The single-crystal structure, electronic structure, and photophysical parameters of these Ru(II) complexes were analyzed and compared. It was found that multinuclear Ru(II) complexes had a pure MLCT (metal-to-ligand charge transfer)–based emissive center, showing longer emission lifetime and higher emission quantum yield, which were desired for oxygen sensing. Then, the oxygen sensing performance of these mononuclear and multinuclear Ru(II) complexes was systematically compared by doping them into polymer fibers *via* electrospinning method. Improved oxygen sensing performance was observed from binuclear Ru(II)-doped nanofibrous samples, compared with the sensing performance of mononuclear ones, including higher sensitivity, shorter response/recovery time, and better photostability. The causation was attributed to the fact that the emissive state of multinuclear Ru(II) complexes was MLCT-based ones and thus more sensitive to O_2_ quenching than monocular Ru(II) complexes whose emissive state was a mixture of MLCT and LLCT (ligand-to-ligand charge transfer). In addition, a multinuclear Ru(II) complex had multiple emissive/sensing components, so that its sensing collision probability with O_2_ was increased, showing better photostability and shorter response/recovery time. The novelty of this work was the linear oxygen sensing curve, which was rarely reported in the previous work.

## 1 Introduction

The research and exploration for functional systems with desired features and performance continuously push the improvement of organic and composite materials (Guan et al., 2015). As a class of important optoelectronic materials, transition metal complexes have shown advantages of adjustable molecular structure, tunable energy level, good solubility, and high stability, which endows them with a wide application in optics, electronics, and related fields (Boens et al., 2012). Emissive Ru(II) complexes coordinated by N-N ligands are nominated as a promising optoelectronic candidate, where N-N means diamine ligands such as phenanthroline and bipyridine (Yao et al., 2014). Theoretical calculation on typical [Ru(N-N)_3_]^2+^ complexes has revealed the electronic structure of MLCT (metal-to-ligand charge transfer) transition, which means that the occupied FMOs (frontier molecular orbitals) are composed of dominant metal d contribution, whereas the unoccupied ones consist of π* orbitals of N-N ligands. Such MLCT-based emissive state generally has emissive lifetime at a scale of microsecond and Stokes shift at a scale of dozens of nanometers, which makes [Ru(N-N)_3_]^2+^ complexes a candidate structural component for optoelectronic materials. For instance, [Ru(N-N)_3_]^2+^ complexes have been uniformly distributed in silica hosts of MCM-41 and SBA-15, serving as an oxygen sensing probe (Lei et al., 2006). In this report, the key sensing parameters of Ru(II)-based probe are greatly improved and comparable with those of Pt(II)-based ones. Keyes and coworkers reported dinuclear Ru(II) complexes for mitochondrial targeted monitoring of dynamic changes to oxygen concentration and ROD generation in live cells (Martin et al., 2014). By doping [Ru(N-N)_3_]^2+^ complexes into a metal-organic framework host, Zhang has constructed singlet oxygen photoreactors, where Ru(N-N)_3_]^2+^ complexes are used as photosensitizer (Zhang et al., 2016). More optical oxygen sensing composites using Ru(N-N)_3_]^2+^ complexes as functional component have been reported (Zhang et al., 2021; Zhao et al., 2021). These results confirm the possibility of using emissive [Ru(N-N)_3_]^2+^ as sensing probe in an oxygen sensing composite.

Previous research efforts have been devoted to the chemical modification of N-N ligands, hoping to improve sensing performance and/or modify features of resulting [Ru(N-N)_3_]^2+^ complexes. For instance, two phenyl rings are introduced into 1,10-phenanthrolin ligand to increase the conjugation size in N-N ligand and thus to increase the sensing collision probability, improving sensing performance (Bian et al., 2021; Zhang et al., 2021). Aiming at a uniform dispersion of [Ru(N-N)_3_]^2+^ probe in a silica-based supporting host and thus avoiding phase separation in supporting host, [Ru(N-N)_3_]^2+^ molecules are covalently grafted into their supporting host. The porous structure of supporting host ensures gradual O_2_ concentration around [Ru(N-N)_3_]^2+^ probe, so that linear sensing response is observed. Besides this silica-based supporting host, some research efforts have reported another type of supporting host of nanofibrous film synthesized by electrospinning (ES) method (Wang et al., 2009; Wang et al., 2010). It was found that ES fibers can be applied as a supporting host for oxygen sensing probe on the basis of emissive metal complex, owing to their uniform and microporous structure that endows sensing probe with better photostability, increased emission lifetime, and restricted structural relaxation in excited state.

The abovementioned research reports, however, are all focused on mononuclear [Ru(N-N)_3_]^2+^ complexes, with little comparison with multinuclear Ru(II) complexes. A tentative causation for the underdeveloped research on multinuclear Ru(II) complexes is that there are multiple emissive centers and thus multiple sensing centers in each sensing site, which compromises the uniform quenching of sensing probe, leading to non-linear working curve (Bian et al., 2021). No further research or solution to this problem has been figured out.

**SCHEME 1:**
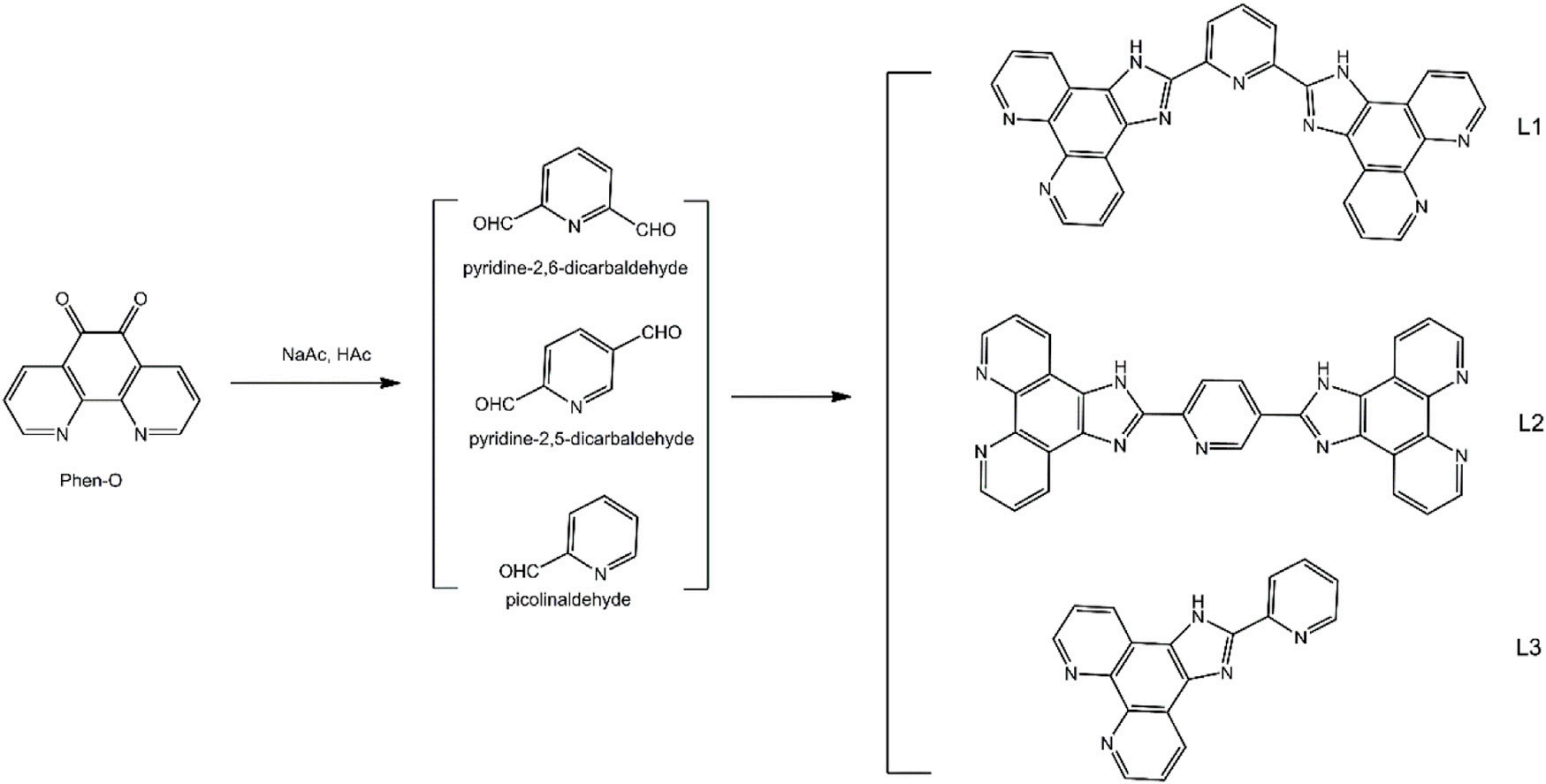
Synthetic protocol for diamine ligands L1, L2, and L3.

Guided by the above consideration, this work focuses on three diamine ligands having two or more chelating sites in each ligand, as well as their [Ru(N-N)_3_]^2+^ complexes using 2,2′-bipyridyl (bpy) as auxiliary ligand, as shown in Scheme 1. A full comparison between multinuclear and mononuclear Ru(II) complexes shall be carried out. By doping them into a supporting host of ES fibers, the influence of inter-molecular aggregation and dispersal in supporting host shall be minimized, so that the sensing performance of multinuclear Ru(II) complexes can be compared with that of mononuclear ones.

## 2 Experimental Section

### 2.1 Equipment and Method

The synthetic protocol for the three diamine ligands (L1, L2, and L3) having two or more chelating sites in each ligand is given in Scheme 1. Their [Ru(N-N)_3_]^2+^ complexes using 2,2′-bipyridyl (bpy) as auxiliary ligand were obtained in accordance with a classic method and then sent to an ES operation to generate [Ru(N-N)_3_]^2+^-doped ES fibers, using poly(styrene) (PS) as host (Wang et al., 2009; Martin et al., 2014). Most chemicals for the synthesis in this work were AR grade ones and supplied by Gongji (Tianjin) chemical reagents cooperation, such as RuCl_3_, 1,10-phenanthroline (Phen), 2,2′-bipyridyl (bpy), PS (Mw = 100,000), pyridine-2,6-dicarbaldehyde, pyridine-2,5-dicarbaldehyde, and picolinaldehyde. Other related chemicals and reagents (AR grade) were purchased from a local chemical regent company (Yuhua Chemical company). NMR spectra were collected on a Bruker Avance 400 spectrometer. MS spectra were recorded by an Agilent 1956B LC/MS spectrometer. Single crystals were analyzed with a Bruker SMART APEX II crystal diffractometer. UV-visible absorption spectra were determined using a Shimadzu UV-3101PC spectrophotometer. Emission spectra and decay dynamics were inspected by a Hitachi F7000 fluorescence spectrometer and an Edinburgh FL920 fluorescence spectrometer excited by a Xe lamp (150 W). Emission quantum yield was determined with this F7000 spectrometer equipped with an integrating sphere, with quinine sulfate (20 μM) in diluted H_2_SO_4_ (0.1 M). Micromorphology was recorded using a Hitachi S4800 microscope. Density functional theory (DFT) calculation was carried out on Ru(II) single crystal with GAMESS at RB3LYP/LANL2DZ level (Yoshikawa et al., 2009; Hasan et al., 2015). Graphical presentation for Ru(II) FMOs was generated with wxMacMolPlot with contour value of 0.025.

### 2.2 Synthesis of Chelating Ligands (L1, L2, and L3)

Chelating ligands (L1, L2, and L3) of this work were synthesized by a classic method using 1,10-phenanthroline-5,6-dione (Phen-O) and aldehydes as starting compounds, in a mixture of HAc and NaAc. First, Phen-O was prepared in accordance with a literature procedure by oxidizing 1,10-phenanthroline in a mixture of concentrated H_2_SO_4_ and HNO_3_ (Lei et al., 2006; Martin et al., 2014). Yellow needles were obtained as Phen-O with yield of 85%. ^1^H NMR (CDCl_3_): δ 9.11 (dd, 2H, J = 4.8 Hz), 8.53 (dd, 2H, J = 6.0 Hz), 7.64 (dd, 2H, J = 4.2 Hz, 6.0 Hz). MS calculated for C_12_H_6_N_2_O_2_, 210.0, MS *m*/*z*: [m]^+^, found 210.04.

L1 (2,6-bis(1H-imidazo[4,5-f][1,10]phenanthrolin-2-yl)pyridine) was prepared in accordance with below method. To a solution of HAc (40 ml) containing NH_4_Ac (13 g), Phen-O (10 mmol) and pyridine-2,6-dicarbaldehyde (5 mmol) were added. The resulting mixture was stirred at 120°C for 13 h under continuous dry N_2_ stream. After natural cooling, cold water (200 ml) was added to yield solid product which was extracted with CH_2_Cl_2_ and purified on an Al_2_O_3_ column. Yellow solid was obtained as L1 with yield of 56%. ^1^H NMR (DMSO-d_6_): δ 7.83–7.85 (m, 4H, J = 4.0), 8.02 (t, 1H, J = 7.2), 8.34 (d, 2H, J = 7.8), 8.93 (d, 4H, J = 3.3), 9.16 (d, 4H, J = 7.6), 14.11 (NH). MS calculated for C_31_H_17_N_9,_ 515.2, MS *m*/*z*: [m]^+^, found 515.15.

L2 (2,2'-(pyridine-2,5-diyl)bis(1H-imidazo[4,5-f][1,10]phenanthroline)) was synthesized following a similar synthetic procedure for L1, except that, in this run, pyridine-2,6-dicarbaldehyde (5 mmol) was replaced by pyridine-2,5-dicarbaldehyde (5 mmol). Yield: 63%. ^1^H NMR (DMSO-d_6_): δ 7.83–7.86 (m, 4H, J = 4.8), 8.01 (t, 1H, J = 7.2), 8.33 (d, 2H, J = 7.8), 8.95 (d, 4H, J = 3.3), 9.02 (m, 2H, J = 7.2), 9.15 (d, 2H, J = 7.6), 14.10 (NH). MS calculated for C_31_H_17_N_9,_ 515.2, MS *m*/*z*: [m]^+^, found 515.16.

L3 (2-(pyridin-2-yl)-1H-imidazo[4,5-f][1,10]phenanthroline) was synthesized following a similar synthetic procedure for L1, except that, in this run, pyridine-2,6-dicarbaldehyde (5 mmol) was replaced by picolinaldehyde (10 mmol). ^1^H NMR (DMSO-d_6_): δ 7.83–7.86 (t, 1H, J = 9.0), 7.90–7.92 (t, 1H, J = 6.0), 8.49–8.55 (dd, 4H, J = 18.0), 8.95 (t, 2H, J = 6.0), 9.09 (s, 2H), 14.14 (NH). MS calculated for C_18_H_11_N_5_, 297.1, MS *m*/z: [m]^+^, found 297.11.

### 2.3 Synthesis of Ru(II) Complexes

The starting compound of Ru(bpy)_2_Cl_2_ was synthesized according to a classic method using bpy as ligand [4,5]. To a redistilled DMF solution (30 ml) containing anhydrous LiCl (60 mmol), RuCl_3_ (10 mmol) and bpy (22 mmol) were added. The resulting mixture was stirred at 120°C for 13 h under continuous dry N_2_ stream. After natural cooling, water ice (300 g) was added to give solid product that was washed with acetone and recrystallized in MeOH to give Ru(bpy)_2_Cl_2_ as dark red solid. Yield: 45%. ^1^H NMR (DMSO-d_6_): δ 7.22–7.25 (d, 4H), 7.73–7.76 (d, 4H), 8.53–8.57 (dd, 4H), 9.15–9.19 (dd, 4H). MS calculated for C_20_H_16_N_4_Cl_2_Ru_1_, 484.0, MS *m*/*z*: [m]^+^, found 483.97.

Ru-1 (stands for [(bpy)_2_Ru(L1)Ru(bpy)_2_]Cl_4_) was synthesized according to a classic method using Ru(bpy)_2_Cl_2_ as starting compound (Lei et al., 2006; Martin et al., 2014). To an ethanol solution (25 ml) containing Ru(bpy)_2_Cl_2_ (5 mmol), L1 (2.5 mmol) was added and stirred at 80 °C for 13 h under continuous dry N_2_ stream. After natural cooling, water ice (100 g) was added to give solid product that was washed with acetone and recrystallized in MeOH to give Ru-1 as red solid. Yield: 71%. ^1^H NMR (DMSO-d_6_): δ 7.36 (t, 4H, J = 6.4), 7.60 (t, 4H, J = 6.5), 7.67 (d, 4H, J = 5.4), 7.88 (d, 4H, J = 5.2), 7.96–8.05 (m, 4H), 8.10–8.15 (m, 8H), 8.25 (t, 4H, J = 7.8), 8.37 (m, 1H), 8.55 (d, 2H, J = 7.1), 8.82–8.86 (m, 8H), 9.12–9.21 (m, 4H), 14.25 (NH). MS calculated for C_71_H_49_Cl_4_N_17_Ru_2_, 1484.1, MS *m*/*z*: [m-Cl_4_]^+^, found 1342.24. Ru-1 single crystal was obtained and analyzed to confirm its molecular structure (CCDC 869272).

Ru-2 (stands for [(bpy)_2_Ru(L2)Ru(bpy)_2_]Cl_4_) was synthesized following a similar synthetic procedure for Ru-1, except that, in this run, L1 (2.5 mmol) was replaced by L2 (2.5 mmol). ^1^H NMR (DMSO-d_6_): δ 7.35 (t, 4H, J = 7.2), 7.62 (t, 4H, J = 6.4), 7.69 (d, 4H, J = 5.4), 7.85 (d, 4H, J = 5.4), 7.99–8.05 (m, 5H), 8.12–8.16 (m, 7H), 8.26 (t, 4H, J = 7.8), 8.36 (m, 1H), 8.57 (d, 2H, J = 7.2), 8.84–8.87 (m, 8H), 9.17–9.21 (m, 4H), 14.23 (NH). MS calculated for C_71_H_49_Cl_4_N_17_Ru_2_, 1484.1, MS *m*/*z*: [m-Cl_4_]^+^, found 1342.25.

Ru-3 (stands for [(bpy)_2_Ru(L3)]Cl_2_) was synthesized following a similar synthetic procedure for Ru-1, except that, in this run, L1 (2.5 mmol) was replaced by L3 (5.0 mmol). Yield: 56%.^1^H NMR (DMSO-d_6_): δ 7.37 (s, 2H), 7.65 (s, 4H), 7.88–7.96 (m, 4H), 8.14 (s, 4H), 8.24 (s, 2H), 8.85–8.87 (d, 4H, J = 6.0), 8.93 (s, 4H), 9.13 (s, 2H), 14.65 (NH). MS calculated for C_38_H_27_Cl_2_N_9_Ru_1_, 783.1, MS *m*/*z*: [m-Cl_2_]^+^, found 711.12. Ru-3 single crystal was obtained and analyzed to confirm its molecular structure (CCDC 1915376).

### 2.4 Synthesis of Ru-n@PS Samples, *n* = 1, 2, 3

Ru(II)-doped nanofibers (Ru-n@PS) in this work were synthesized in accordance with a classic ES method, using PS as supporting host (Wang et al., 2009). PS was firstly weighted and then stirred in DMF to form a bubble-free solution (20 wt%), and then, Ru-n (*n* = 1, 2, 3) was weighted and mixed with this DMF solution. Three doping levels were tried for each Ru-n complex, including 10, 12, and 15 wt%, respectively. Then, these solutions were sent for ES operation. A plastic syringe equipped with a nozzle was connected with the anode terminal of a high-voltage power supply. A piece of Al foil (5 × 5 cm) served as the collecting plate that was connected with ground electrode. The driving voltage was set as 18 kV and the distance between nozzle tip and collecting plate was 30 cm.

### 2.5 Evaluation of O_2_ Sensing Behavior

The oxygen sensing behavior of Ru-n@PS was tentatively discussed on the basis of steady emission spectra. Solid Ru-n@PS was loaded in a quartz chamber. Surrounding atmosphere was adjusted by mixing dry N_2_ and O_2_ streams *via* gas meters and then imported in the quartz chamber. Steady emission spectra of Ru-n@PS were recorded after 5 s of each atmospheric adjustment.

## 3 Results and Discussion

### 3.1 Geometric Structure of Ru-N Single Crystals

**SCHEME 2:**
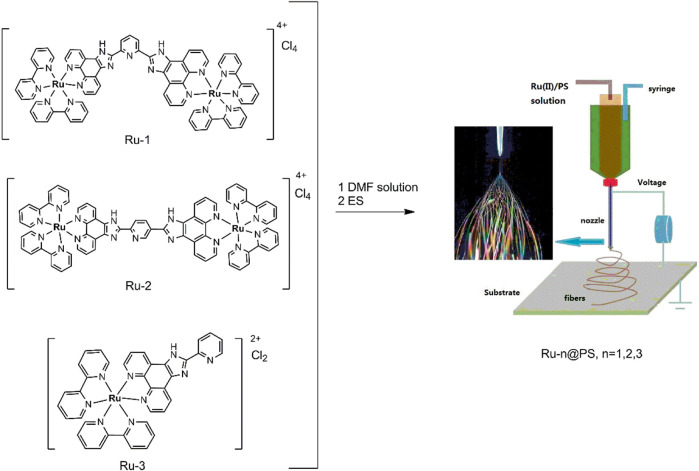
Preparation route for Ru-n@PS fibers.

**FIGURE 1:**
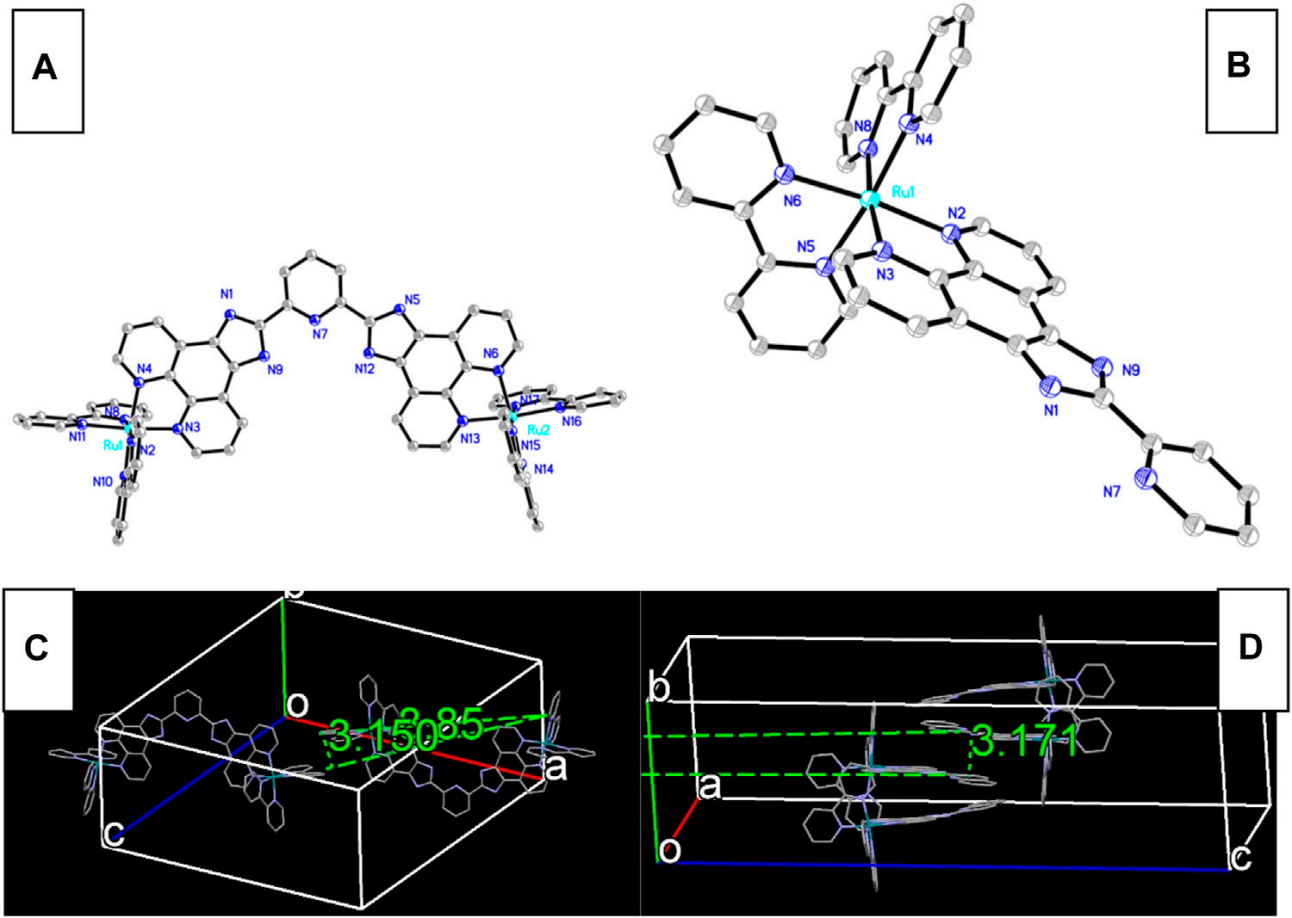
Single-crystal structure of Ru-1 **(A)** and Ru-3 **(B)**, and their packing mode [**(C)** for Ru-1 and **(D)** for Ru-2].

As shown in Scheme 1 and Scheme 2, there are electron-deficient coplanar π planes in L1, L2, and L3 and their Ru(II) complexes, so that Ru-n (*n* = 1, 2, 3) molecules have a rigid structure, which favors the formation of their single crystals. The single-crystal structures of Ru-1 and Ru-3 were luckily obtained (CCDC 869272 and 1915376) and shown in Figure 1, where the large conjugation planes in them are clearly observed. According to literature reports, the unoccupied FMOs of MLCT-based sensing probes consist of π* of diamine ligands (Yao et al., 2014; Lei et al., 2006; Martin et al., 2014). A large conjugation plane in diamine ligand shall certainly enhance the collision probability between O_2_ quencher and excited MLCT electrons, showing better sensing performance. A monoclinic system is adopted by both Ru-1 and Ru-3 crystals, where each Ru(II) coordination center falls in the center of an octahedral coordination sphere formed by three bidentate diamine ligands. Regardless of the multiple coordination sites in L1, which are the one formed by two Phen N atoms and the one formed by 3 N atoms from one pyridine and two benzimidazole groups, Ru(II) coordinates with the phenanthroline N atoms only. A possible explanation is that the 3-N-atom coordination site has large steric hindrance and thus is not adopted by Ru(II) ions. On the other hand, the Phen-N-atom coordination site is fully available for Ru(II) ions, so that L1 forms a binuclear Ru(II) complex (Ru-1), instead of a triple nuclear one.

Some key structural parameters of these coordination sites in Ru-1 and Ru-3 are selected and shown in Supplementary Table S1 (Supporting Information). These coordination sites are octahedral ones, with geometric distortion caused by Jahn-Teller effect (Toriyyama et al., 2004). Their Ru(II)-N bond length values fit in a restricted region of 2.06 ± 0.03 Å, whereas the ligand bite angles with Ru(II) center localize in a region of 79 ± 2°. These values are similar to those of literature octahedral Ru(II) crystals with diamine ligands (Yao et al., 2014). The Ru-N bond length and N-Ru-N bond angle values of binuclear Ru-1 are comparable to corresponding values of mononuclear Ru-3, which means that L1 owns a similar coordination affinity toward Ru(II), compared with L3, regardless of the difference between their coordination site amount. This observation ensures a fair comparison between Ru-1 and Ru-2 sensing performance later.

There is a disadvantage with these rigid and coplanar conjugation planes, though. It is observed from Figure 1 that the π-π attraction between these coplanar rigid planes makes Ru-n molecules align orderly with each other, showing inter-molecular stacking. As for Ru-1, a bpy ring from a Ru-1 molecule aligns nearly parallel to another bpy ring from another Ru-1 molecule with distance of 3.15 Å and interception angle of 8.85°. As for Ru-3, four Ru-3 molecules are aggregated together with a typical distance of 3.17 Å and interception angle of 0.0°. Such inter-molecular stacking has been proved as a good factor for luminescent materials because it effectively suppresses the geometric relaxation that occurs in complex excited state (Zhang et al., 2009). It is, however, not favored for oxygen sensing. Regardless of the blue shifted emission, prolonged emission lifetime, and improved emission intensity caused by such inter-molecular stacking, it brings a serious steric barricade to oxygen sensing collision and thus compromises sensing performance badly, leading to long response time and unsatisfactory sensitivity. This issue may be solved by dispersing Ru-n molecules into a proper host, which will be discussed later.

### 3.2 Electronic Configuration of Ru-N by DFT Calculation

**FIGURE 2:**
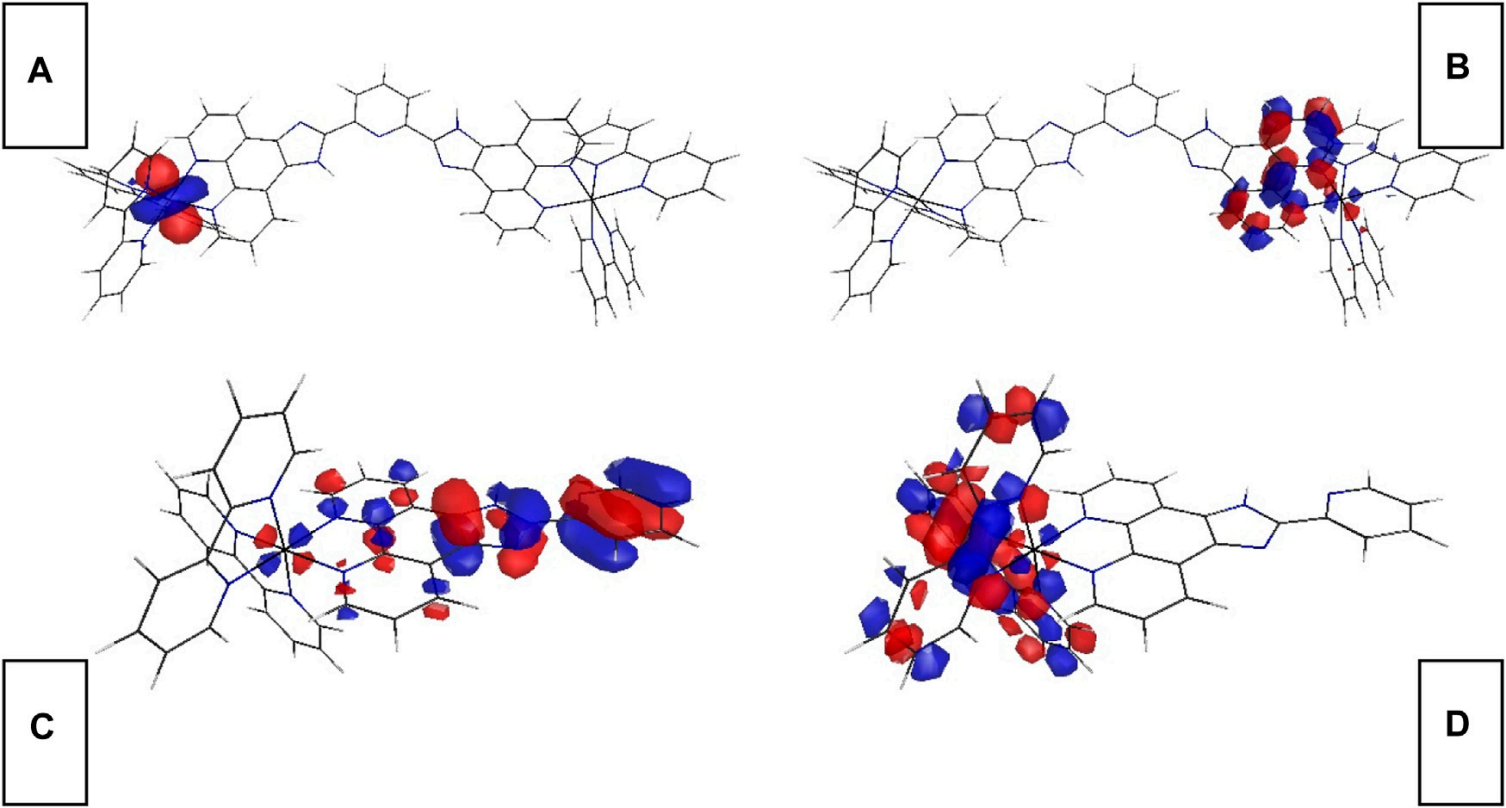
Graphical presentation for Ru-n FMOs. **(A)** Ru-1 HOMO, **(B)** Ru-1 LUMO, **(C)** Ru-3 HOMO, and **(D)** Ru-3 LUMO.

Aiming at a better understanding on the electronic configuration of Ru-n complexes, DFT calculation is performed on Ru-1 and Ru-3 single-crystal structure. Corresponding FMOs are plotted in Figure 2. As for the binuclear Ru(II) complex (Ru-1), its occupied FMO (HOMO) consists of metal d contribution from one of its two metal centers. Its unoccupied FMO (LUMO) is mainly composed of Phen component in L1 ligand, in addition to the contribution from metal d contribution. In this case, the onset electronic transition of Ru-1 from HOMO to LUMO shall be assigned as a mixed character of MRuL_1_CT (metal-to-L1-charge transfer) and MRuCCT (metal-centered charge transfer). A similar case is observed for the mononuclear Ru(II) complex (Ru-3). Ru-3 HOMO mainly consists of benzimidazole ring from ligand L3, along with some contribution from Ru(II) d orbitals. As a consequence, the onset electronic transition of Ru-3 from HOMO to LUMO shall be assigned as a mixed character of M_Ru_L_3_CT (metal-to-L3 charge transfer), L_3_L_bpy_CT (L3-to-bpy charge transfer), and M_Ru_CCT (metal-centered charge transfer). Generally, MCCT transition should be avoided in emitters on the basis of luminescent metal complexes because a metal d-d transition is a forbidden one by transition selection law, which consequently brings negative effects to luminescence, including decreased emissive probability, low emission quantum yield, and fast emission dynamics. No surprise that these negative effects compromise oxygen sensing performance as well because they shrunk the collision probability between excited sensing probe and O_2_ molecules. There is more Ru(II) d-d participation in Ru-3 FMOs, compared with Ru-1 FMOs. The Ru(II) d-d participation effect on oxygen sensing performance will be discussed in detail later.

The onset electronic transition (S_0_→S_1_) energy value of Ru-1 is 2.4892 eV, which is rather similar to that of Ru-3 (2.4791 eV). Although with a large coplanar conjugation plane in L1, Ru-1 still has a higher S_0_→S_1_ energy value than Ru-3. This finding seems in confliction with a literature conclusion that states that an enlarged conjugation chain in diamine ligand of luminescent metal complexes shall decrease singlet excitation energy by lifting their HOMO level and decreasing LUMO level (Zhang and Li, 2009). In this work, the electron-deficient L1 ligand neutralizes the effect of large conjugation plane on decreasing excitation energy.

### 3.3 Morphology of Ru-n@PS Fibers: SEM, Elemental Mapping, and Fluorescence Microscopy Images

**FIGURE 3:**
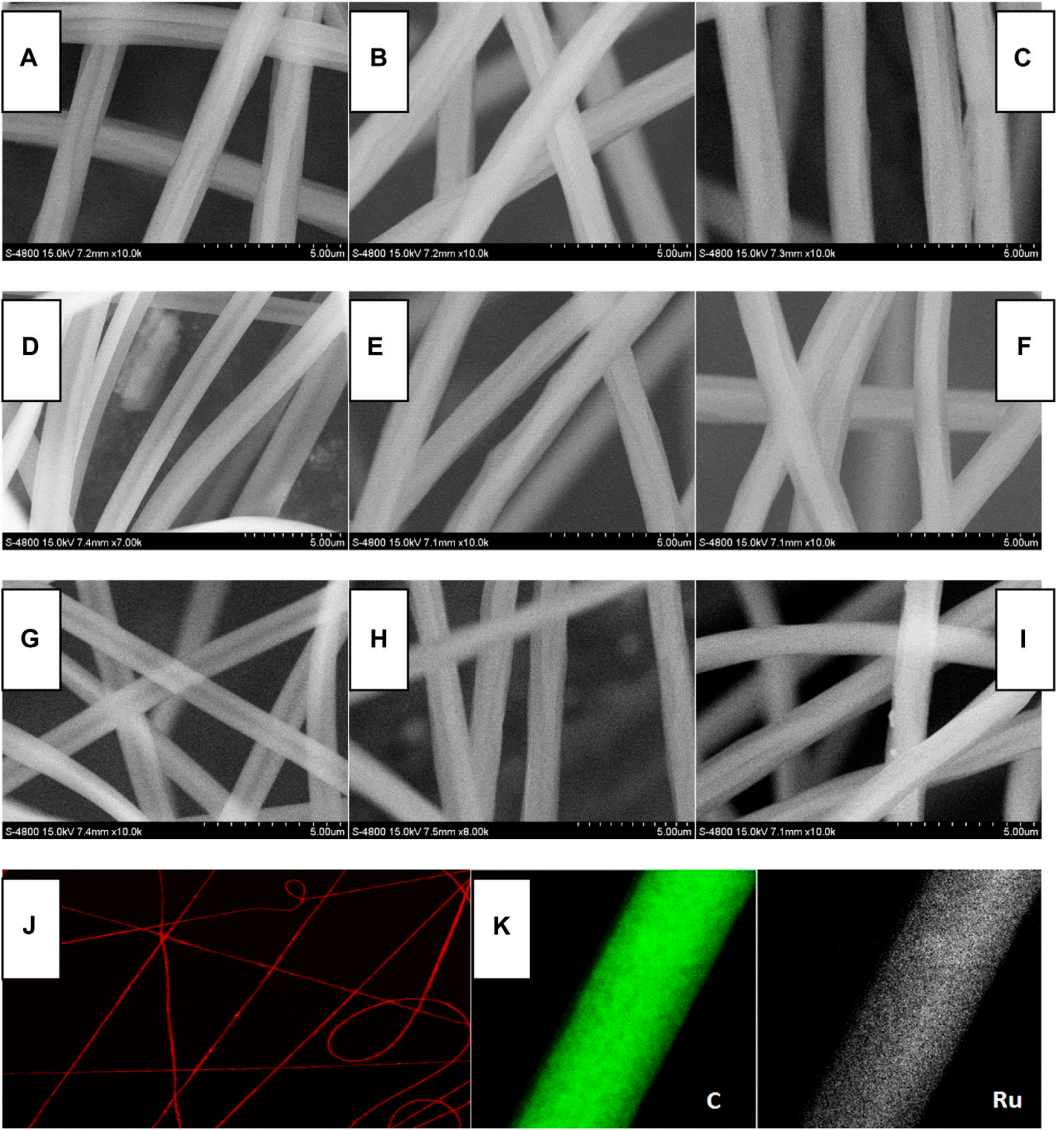
SEM images of Ru-n@PS fibers. **(A)** Ru-1@PS(10%), **(B)** Ru-1@PS(12%), **(C)** Ru-1@PS(15%), **(D)** Ru-2@PS(10%), **(E)** Ru-2@PS(12%), **(F)** Ru-2@PS(15%), **(G)** Ru-3@PS(10%), **(H)** Ru-3@PS(12%), and **(I)** Ru-1@PS(15%). Fluorescence microscopy image **(J)** and EDX mapping **(K)** of Ru-1@PS(15%).

The inter-molecular stacking of Ru-1 and Ru-3 has been observed in Section 3.1, which is considered as a negative factor for oxygen sensing. To eliminate such inter-molecular stacking and their potential negative effect on oxygen sensing performance, Ru-n complexes are dispersed and immobilized into a PS supporting matrix by ES technique. Three doping levels were tried for each Ru-n complex, aiming at performance optimization, including 10, 12, and 15 wt%, respectively. The scanning electron microscopy (SEM), fluorescence microscopy, and elemental mapping images of Ru-n@PS nanofibers shown in Figure 3 offer a direct evaluation on their morphology. Random and orderless distribution is observed for these nanofibers. All nanofibers align cross each other on their substrates, resulting in a tight but porous microstructure that is O_2_-penetratable. In this case, an effective sensing collision between Ru-n sensing probe and O_2_ molecules can be expected, showing sensing behavior with fast response and complete quenching. These nanofibers have shown smooth and homogeneous surface. They are similar to each other in their mean diameter (∼1 μm), regardless of various Ru-n complexes and their doping levels. There seems no direct correlation between the morphology of Ru-n@PS and dopant kind/doping level, indicating a good compatibility between PS host and Ru-n dopant. It is observed from Figure 3 that Ru element is uniformly distributed across the whole Ru-n@PS fibers, with no intense aggregation, no phase separation, no knot, or no branch structure. Such homogeneous Ru(II) distribution in Ru-n@PS confirms that there is no inter-molecular stacking in these nanofibers, which favors later sensing application, as abovementioned.

### 3.4 Photophysical Parameters of Ru-n and Ru-n@PS Fibers

#### 3.4.1 Absorption and Excitation Spectra

**FIGURE 4:**
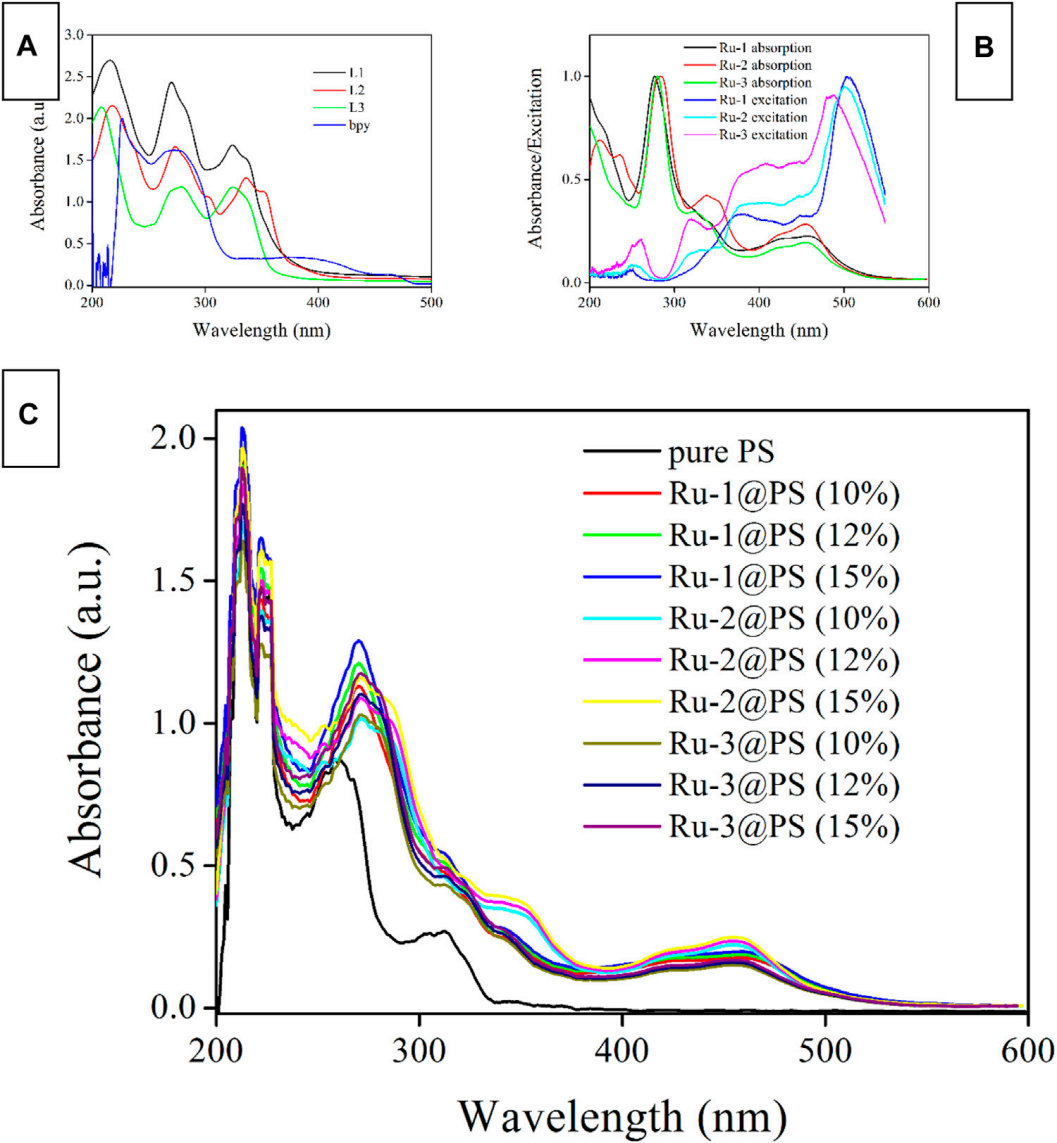
Absorption and excitation spectra of ligands **(A)** and Ru-n complexes **(B)** in DMF (1 μM), and absorption spectra of Ru-n@PS fibers **(C)** in the solid state.

As shown in Figure 4, the absorption spectra of Ru-n are recorded and compared with those of ligands (L1, L2, L3, and bpy). It is observed that bpy absorption falls in high-energy UV region ranging from 230 to 310 nm. Considering their high molar extinction coefficient, these absorption bands are assigned as spin-allowed π-π* electronic transitions. Similarly, the strong absorption bands of diamine ligands (L1, L2, and L3) ranging from 210 to 370 nm are assigned as spin-allowed π-π* electronic transitions. These diamine ligands share rather similar molecular structures, which explains their nearly identical absorption spectra. Only minor absorption redshift is observed for L2, compared with L1 and L3, owing to the large coplanar conjugation plane in L2. After coordinating with Ru(II) center, the abovementioned absorption peaks are all traced from the absorption spectra of Ru-1, Ru-2, and Ru-3. No obvious spectral shift is observed, compared with the absorption spectra of L1, L2, and L3, which means that the ligand spin-allowed π-π* electronic transitions have been preserved in Ru-1, Ru-2, and Ru-3. A weak and broad absorption band ranging from 380 to 510 nm is observed for each Ru-n (*n* = 1, 2, 3) complex, which has not been observed for any ligands. It is regarded as electronic transitions of M_Ru_LCT and LLCT (ligand-to-ligand charge transfer), according to the abovementioned DFT calculation result.

The excitation spectra of Ru-n (*n* = 1, 2, 3) complexes shown in Figure 4 suggest that these weak electronic transitions of M_Ru_LCT and LLCT are rather effective in exciting Ru-n emissive center, despite their weak absorption coefficients. The optimal excitation region for Ru-n (n = 1, 2, 3) complexes falls in low-energy region from 380 to 530 nm. For excitation wavelength longer than 530 nm, the low absorption coefficient compromises excitation intensity, which leads to a cutoff of excitation spectra at ∼550 nm. Whereas the high-energy region ranging from 200 to 350 nm is rather ineffective in exciting Ru-n emissive center. Indeed, ligand spin-allowed π-π* electronic transitions are powerless in exciting Ru-n emissive center because ligand excited state has to experience a series of geometric distortion and potential surface crossing procedures before finally achieving the MLCT-based emissive center, which greatly limits their excitation efficiency (Qiao et al., 2019; Wang et al., 2017; Amao et al., 2000). As for MRuLCT and LLCT transitions, they are able to transfer their energy directly to the MLCT-based emissive center of Ru-n with a high efficiency. Similar to the case of Ru-n absorption, these three excitation spectra are nearly identical to each other, owing to the rather similar molecular structures of Ru-n.

Ru-n (*n* = 1, 2, 3) complexes have preserved their characteristic absorption bands when they are immobilized in PS host, as shown in Figure 4. All absorption bands have been traced with no obvious spectral shift or no new bands. The absorption spectra of Ru-n@PS fibers are considered as the absorption combination between PS host and Ru-n complexes. There shall be only weak interaction between Ru-n dopant molecules and PS chains. In other words, Ru-n molecules are merely immobilized and dispersed by PS framework, forming a porous and O_2_-penetratable structure for Ru-n probe, so that oxygen sensing behavior can be expected.

#### 3.4.2 Emission Spectra

**FIGURE 5:**
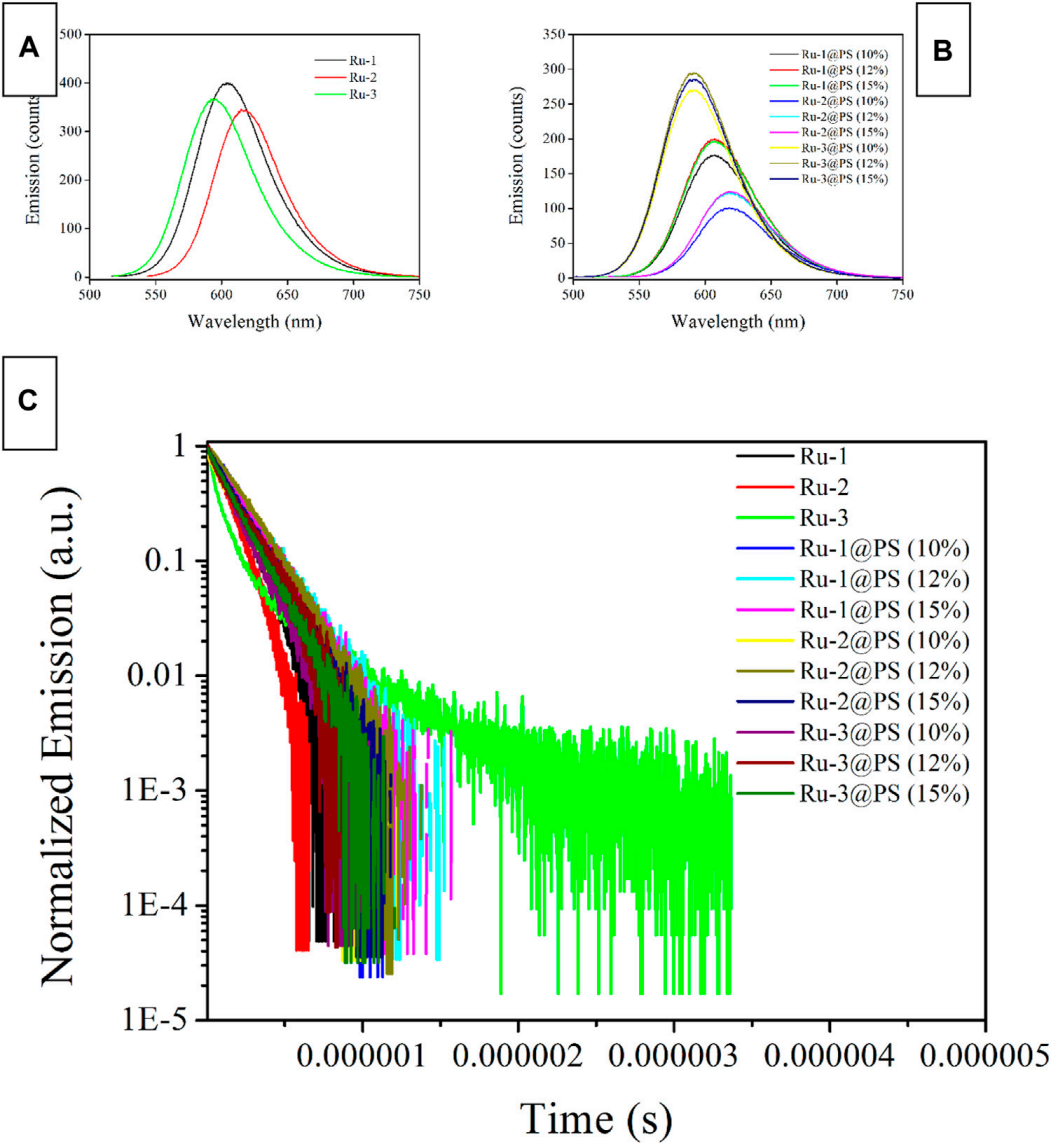
Emission spectra **(A, B)** and emission decay dynamics **(C)** of Ru-n complexes and Ru-n@PS fibers in the solid state.

For comparison convenience, the emission spectra of Ru-n complexes and Ru-n@PS fibers are recorded and shown in Figure 5. Typical Gaussian-shaped emission bands are observed for all samples, including binuclear ones. No dual emission bands are observed for binuclear Ru(II) complexes (Ru-1 and Ru-2), indicating that their two emissive centers localize at the same degenerate level, so that their emissive energy values are exactly the same to each other. These emission bands are broad ones with no vibrational progressions; this finding matches their CT-based emissive center (Amao et al., 2000; Zheng et al., 2011; Guan et al., 2014; Zheng et al., 2003). Aiming at a full comparison between Ru-n complexes and Ru-n@PS fibers, their photophysical parameters, such as emission maxima (λ_em_), full width at half maximum (FWHM), emission decay dynamics (τ), and emission quantum yield (Φ), are summarized in Table 1. L2 exhibits the largest size of coplanar conjugation plane among three diamine ligands, so that Ru-2 exhibits the lowest emissive energy peaking at 614 nm. Correspondingly, the highest emissive energy of Ru-3 peaking at 592 nm is explained by the smallest size of coplanar conjugation plane in its diamine ligand (L3). Ru-2 has the smallest FWHM value of 59 nm, compared with those of Ru-1 (63 nm) and Ru-3 (62 nm). This observation is tentatively explained by its symmetric molecular structure that accelerates emissive decay of emissive center (Zheng et al., 2003).

**TABLE 1:** Photophysical parameters of Ru-n complexes and Ru-n@PS fibers in the solid state.

Sample	λ_em_ (nm, ±1 nm)	FWHM (nm, ±1 nm)	Τ (μs, ±5%)^a^	Φ^a^ (±5%)	k_r_ (10^6^s^−1^)	k_nr_ (10^6^s^−1^)
Ru-1	603	63	0.14	0.27	1.93	5.21
Ru-2	614	59	0.12	0.25	2.08	6.25
Ru-3	592	62	0.10^b^	0.29	2.90	7.10
Ru-1@PS(10%)	607	64	0.20	0.29	1.45	3.55
Ru-1@PS(12%)	607	64	0.21	0.31	1.48	3.29
Ru-1@PS(15%)	607	64	0.21	0.30	1.43	3.33
Ru-2@PS(10%)	618	64	0.18	0.27	1.50	4.05
Ru-2@PS(12%)	618	65	0.21	0.32	1.52	3.24
Ru-2@PS(15%)	618	64	0.18	0.29	1.61	3.94
Ru-3@PS(10%)	591	64	0.15	0.33	2.20	4.46
Ru-3@PS(12%)	593	64	0.18	0.35	1.95	3.61
Ru-3@PS(15%)	593	65	0.16	0.34	2.12	4.13

a Determined under N_2_ stream.

b Single exponential decay pattern is applied for all samples, except for Ru-3. Biexponential decay pattern is only applied for Ru-3, with *τ*_1_ = 0.05 μs and *τ*_2_ = 0.21 μs; here, a weighed mean value (0.10 μs) is used.

As for Ru-n@PS fibers, their emission bands are further broadened and redshifted. This is because PS chains offer a rigid and restricted microenvironment around Ru-n molecules, so that the geometric relaxation of MLCT excited state can be suppressed, which consequently leads to stabilized emissive center, showing prolonged emission decay dynamics, decreased emissive energy, and emission redshift. On the other hand, the Gaussian-shaped emission band is preserved by each sample, with no new emission bands, no shoulder peaks, or no vibrational progressions. It is thus confirmed that the MLCT-based emissive center of Ru-n dopant has been preserved after being doped into PS host. It is still observed that a minor emission redshift is accompanied by increasing doping level, which shall be attributed to the self-aggregation between Ru-n molecules at high doping level.

#### 3.4.3 Emission Dynamics and Quantum Yield

Aiming at a systematical comparison between Ru-n complexes and Ru-n@PS fibers, corresponding emission dynamics (τ) and quantum yield (Φ) values are listed in Table 1. Given pure N_2_ atmosphere, only Ru-3 follows biexponential decay pattern, whereas the other two Ru-n complexes adopt single exponential decay pattern, as shown in Figure 5. For most mononuclear Ru(II) complexes having diamine ligands, biexponential decay pattern is usually adopted, except for Ru(II) polypyridyls in solution, which are usually monoexpoential decay (Yao et al., 2014, Zhang et al., 2009). Such biexponential decay dynamics suggest a potential surface crossing procedure among two emission decay centers. Combined with DFT calculation on Ru-3 mentioned in Section 3.2, L_3_L_bpy_CT dynamic decay shall be responsible for the short-lived decay component, whereas M_Ru_L_3_CT shall be responsible for the long-lived one. The strong UV-visible absorption of Ru-3 tentatively confirms this assignment. The monoexponential decay pattern of Ru-1 and Ru-2 shall be named as the decay dynamics of M_Ru_L_1/2_CT, considering their long decay lifetimes. It seems that the L_1/2_L_bpy_CT dynamic decay has been effectively decreased, showing monoexponential decay pattern. Apparently, this is because there are two emissive centers in degenerate state in Ru-1 and Ru-2, which outnumber L_3_L_bpy_CT dynamic decay.

The Φ values of three Ru-n complexes and Ru-n@PS fibers are recorded and listed in Table 1. The mononuclear Ru-3 shows the highest Φ value among three Ru-n complexes, indicating that the two emissive centers in degenerate state of Ru-1 and Ru-2 are no better than the mono emissive center in Ru-3. As for Ru-n@PS samples, their τ and Φ values are obviously higher than corresponding ones of their dopant, which means that dopant photoluminescence performance has been improved after being immobilized by PS host. It is assumed that PS chains offer a rigid and restricting microenvironment around dopant molecules, which limits structural relaxation in excited sate, showing improved τ and Φ values. This hypothesis is confirmed by analyzing their radiative and non-radiative decay probability constants (k_r_ and k_nr_), using Formulas 1 and 2.

$$\tau =1/\left(k_{r}+k_{\mathrm{nr}}\right)$$ (1)

$$\Phi =k_{r}/\left(k_{r}+k_{\mathrm{nr}}\right)$$ (2)

As shown in Table 1, the radiative probability constant (k_r_) value of Ru-3 is bigger than those of Ru-1 and Ru-2. As abovementioned, the emission decay dynamics of Ru-3 is composed of L_3_L_bpy_CT and M_Ru_L_3_CT, the former decay is a fast one, and the latter one is a slow one. The emission decay dynamics of Ru-1 and Ru-2 is pure M_Ru_L_1/2_CT and thus has a low k_r_ value because metal-related transitions have limited oscillator strength (Zhang et al., 2009). As for Ru-n@PS samples, their k_r_ values are found obviously lower than those of their dopants. A possible explanation is that dopant molecules are immobilized by PS chains, so that their free rotation and vibration are suppressed. Because such rotation and vibration are needed for an excited state to cross potential surface and achieve the lowest excited state, it is rational to see the lower k_r_ values of Ru-n@PS fibers. This hypothesis also explains why Ru-3@PS samples follow single exponential decay pattern, instead of biexponential pattern. Apparently, the short-lived L_3_L_bpy_CT procedure of Ru-3 is suppressed by PS rigid framework. Meanwhile, it is observed that the k_nr_ values of Ru-n@PS samples are obviously lower than those of their dopants. This observation means that PS rigid chains literally control and limit the geometric relaxation in MLCT excited state. In other words, both k_r_ and k_nr_ values are decreased by PS immobilization effect.

### 3.5 O_2_ Sensing Performance of Ru(II)-Doped PS Fibers

#### 3.5.1 Emission Spectra Upon Different O_2_ Concentrations

**FIGURE 6:**
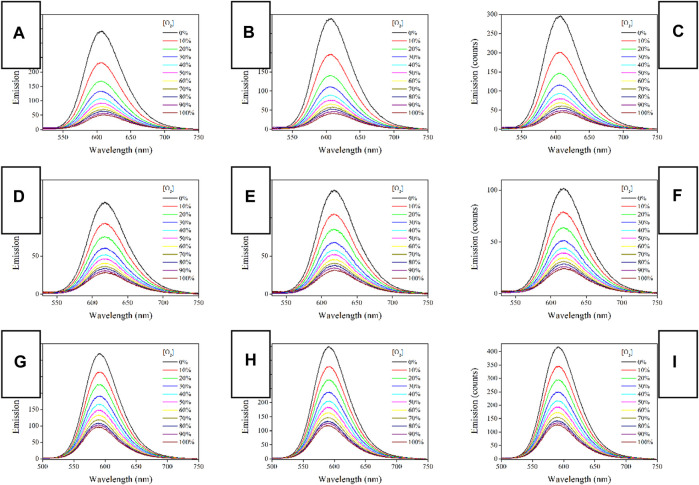
Emission spectra of Ru-n@PS fibers under increasing oxygen concentrations from 0 to 100%. **(A)** Ru-1@PS(10%), **(B)** Ru-1@PS(12%), **(C)** Ru-1@PS(15%), **(D)** Ru-2@PS(10%), **(E)** Ru-2@PS(12%), **(F)** Ru-2@PS(15%), **(G)** Ru-3@PS(10%), **(H)** Ru-3@PS(12%), and **(I)** Ru-1@PS(15%).

It has been abovementioned that all three Ru-n complexes have shown long-lived emission decay dynamics, making them quenchable by O_2_ molecules. To eliminate inter-molecular stacking and their potential negative effect on oxygen sensing performance, Ru-n complexes are dispersed and immobilized into a PS supporting matrix by ES technique, forming a porous and O_2_-penetrable structure. As a consequence, oxygen sensing behavior is desired from Ru-n@PS fibers. As a start, their emission spectra upon different O_2_ concentrations are recorded and compared in Figure 6, so that their oxygen sensing performance can be tentatively discussed. Each Ru-n@PS sample decreases its emission intensity with increasing O_2_ concentration. Meanwhile, the Gaussian-shaped emission band is well preserved, including both mononuclear and binuclear Ru(II) complexes, which means the preservation of MLCT-based emissive center. A dynamic sensing between Ru-n and O_2_ molecules is thus presumed, following literature conclusions (Lei et al., 2006; Yao et al., 2014). Here, the excited Ru(II) probe interacts with O_2_ ground state and loses its energy, showing emission quenching behavior toward O_2_. This dynamic quenching mechanism is tentatively confirmed by the lifetime comparison between pure N_2_ and pure O_2_ conditions. As shown in Supplementary Figure S1 (Supporting Information), monoexponential decay pattern is well preserved, with lifetime greatly decreased to 0.07 μs under pure O_2_ condition. This observation follows a dynamic quenching mechanism. Aiming at a systematical comparison on sensing performance of Ru-n@PS, sensing sensitivity is defined as the value of I_0_/I_100_. Here, I_0_ and I_100_ stand for the emission peak intensity values of O_2_ concentration of 0% and 100%, respectively. As listed in Table 2, the sensitivity values of Ru-1@PS and Ru-2@PS samples are higher than those of Ru-3@PS, suggesting that binuclear Ru(II) complexes are superior to mononuclear Ru(II) complex in sensitivity. We attribute the causation to the long τ values of Ru-1 and Ru-2, compared with that of Ru-3. Apparently, there will be more sensing collision chances between probe and O_2_ molecules for a sensing probe with a long excited sate lifetime. It has been abovementioned that the emission decay dynamics of Ru-3 is composed of L_3_L_bpy_CT and M_Ru_L_3_CT. Only the latter one is a slow one and O_2_-sensitive, whereas the former decay is a fast one that has less sensing collision chances with O_2_ molecules. The emission decay dynamics of Ru-1 and Ru-2 is pure M_Ru_L_1/2_CT and thus has more sensing collision chances with O_2_ molecules, showing higher sensitivity values.

**TABLE 2:** Sensing and fitting parameters of Ru(II)@PS fibers.

Dopant, concentration	*I*_0_/*I*_100_	K_sv_ (O_2_%^-1^)	R^2^	T_res_ (s)	T_rec_ (s)	References
Ru-1@PS(8%)	6.72±0.02	0.057±0.002	0.9989	15±1	30±2	This work
Ru-1@PS(10%)	6.94±0.02	0.059±0.002	0.9986	15±1	30±2	This work
Ru-1@PS(12%)	6.47±0.02	0.055±0.002	0.9991	15±1	30±2	This work
Ru-2@PS(8%)	4.25±0.01	0.032±0.001	0.9995	15±1	28±2	This work
Ru-2@PS(10%)	4.39±0.01	0.033±0.001	0.9993	15±1	28±2	This work
Ru-2@PS(12%)	4.24±0.01	0.032±0.001	0.9996	15±1	28±2	This work
Ru-3@PS(8%)	3.29±0.01	0.024±0.001	0.9989	6±1	29±2	This work
Ru-3@PS(10%)	3.35±0.01	0.024±0.001	0.9991	6±1	29±2	This work
Ru-3@PS(12%)	3.27±0.01	0.022±0.001	0.9988	6±1	29±2	This work
[Cu(POP)ohencarz]BF_4_-PS	15.56	0.1492	0.9966	∼8	∼14	Wang et al. (2009)
[Cu(dpb)(dmp)]PF_6_-PS	1.7	Non-linear	N/A	∼24	∼40	Guan et al. (2014)
[Cu(POP)(PTZ)]BF_4_-MCM-41	11.16	Non-linear	0.9967	4	78	Zhang et al. (2021)
[Cu(dpephos)(enip)]BF_4_-MCM-41	5.95	Non-linear	N/A	8	39	Bian et al. (2021)
Ru thin film	∼5.81	Non-linear	0.1249	∼4	∼10	Lei et al. (2006)
Ru bulk	∼14.31	Non-linear	0.1369	∼4	∼10	Lei et al. (2006)
Ru/SBA-15	13.89	Non-linear	0.2821	∼4	∼27.5	Martin et al. (2014)
Ru-MCM-41	20.48	Non-linear	0.2750	13.5	22.0	Martin et al. (2014)

The sensitivity values of Ru-1@PS samples are higher than those of Ru-2@PS samples, although the τ and electronic configuration of Ru-1 are rather similar to those of Ru-2. We attribute the causation for the sensitivity difference between Ru-1@PS and Ru-2@PS to the geometric factor. As for Ru-2, its linearly symmetric structure puts two emissive/sensitive centers opposite to each other. In this case, each emissive/sensitive center is covered by the other one, bringing steric hindrance to O_2_ attack and thus compromising sensing behavior; Whereas, the two emissive/sensitive centers of Ru-1 are distributed non-linearly, which decreases the steric hindrance for O_2_ attack, resulting in a higher sensitivity.

#### 3.5.2 Working Plots and Stern-Volmer Fitting

The above emission spectra of Ru-n@PS upon different O_2_ concentrations are analyzed with Stern-Volmer equation (Lei et al., 2006; Martin et al., 2014). Assuming that probe molecules are localized in a homogeneous matrix, if they follow dynamic sensing mechanism, then the Stern-Volmer equation of emission intensity form will be described by Formula 3. Here, I_0_ denotes the emission intensity in the absence of any quencher, I means emission intensity, Ksv is Stern-Volmer fitting constant, and [O_2_] is oxygen concentration, respectively. I_0_/I = 1 + K_SV_[O_2_] Formula 3.

**FIGURE 7:**
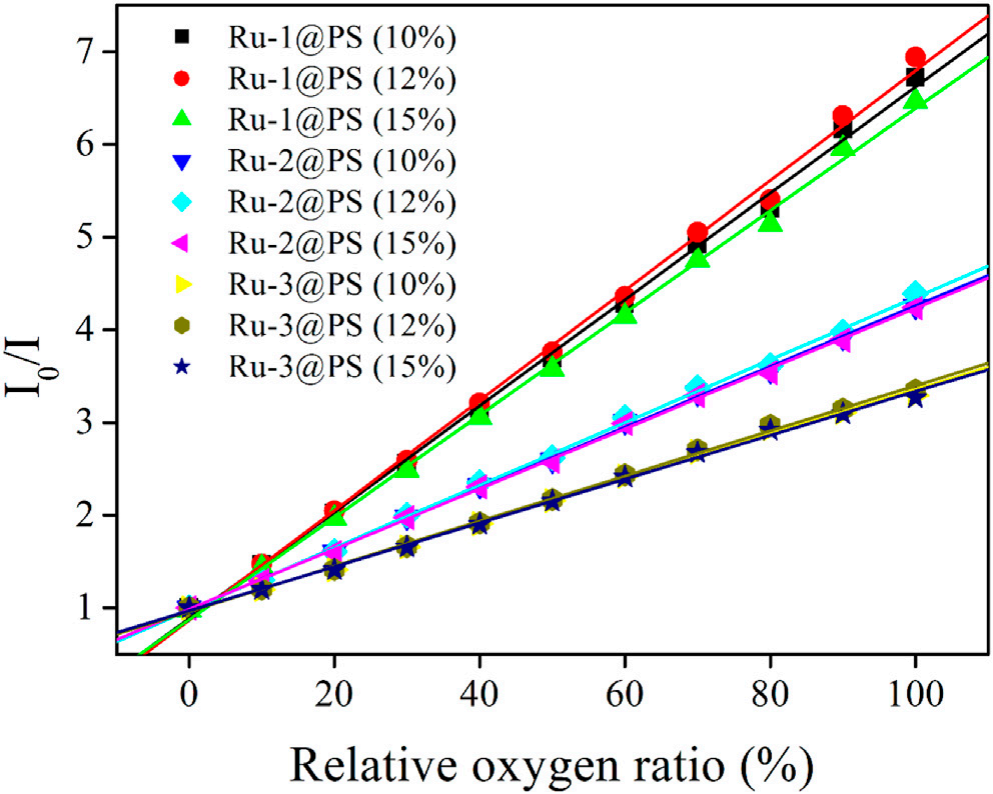
Stern-Volmer plots of Ru-n@PS fibers under increasing oxygen concentrations from 0% to 100%.

Considering that the uniform dispersal of Ru-n in PS host has been confirmed in Section 3.3 and that the dynamic sensing of Ru-n toward O_2_ has been approved in Section 3.5.1, Formula 3 shall be applied for the emission spectra of Ru-n@PS upon different O_2_ concentrations. As shown in Figure 7, all Ru-n@PS working plots fit linear response toward various O_2_ concentrations. Corresponding fitting parameters are shown in Table 2. Non-linear sensing response is usually observed for most Ru(II)-based sensing systems, owing to their biexponential decay dynamics. Owing to their rather different decay dynamics, corresponding emissive components shall not be uniformly quenched, resulting in non-linear sensing behavior. In this work, Ru-n@PS samples have shown unified MLCT-based monoexponential decay dynamics, which leads to linear sensing response. With these linear working curves, the practical sensing performance of a typical Ru-n@PS sample is recorded and shown in Supplementary Table S2 (Supporting Information). Positive errors of ∼2%–4% are observed.

#### 3.5.3 Response/Recovery Time and Photostability

**FIGURE 8:**
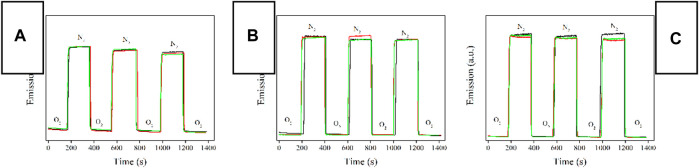
Emission intensity monitoring of Ru-n@PS fibers upon surrounding atmosphere periodically switched between pure N_2_ and pure O_2_. Blank means 10%, red means 12%, and green means 15%. **(A)** Ru-1@PS, **(B)** Ru-2@PS, and **(C)** Ru-3@PS.

To get a direct confirmation on Ru-n@PS emission intensity variation caused by O_2_ presence, Ru-n@PS emission intensity is continuously monitored with surrounding atmosphere periodically switched between pure N_2_ and pure O_2_. As shown in Figure 8, under pure N_2_ atmosphere, Ru-n@PS emission intensity is strong and well preserved, showing a good photostability. When the surrounding atmosphere is switched to pure O_2_, Ru-n@PS emission intensity is decreased instantly and then preserved, showing oxygen sensing behavior. By switching the surrounding atmosphere back to pure N_2_, Ru-n@PS emission intensity is recovered to its original level and then preserved. Generally speaking, all three Ru-n@PS samples have shown good photostability, no matter what kind of dopant is applied, mononuclear (Ru-3) or binuclear ones (Ru-1 and Ru-2). The observation of such good photostability is attributed to the protecting effect from PS host. A systematical comparison between Ru-n@PS samples on their response and recovery behavior toward O_2_ is performed by analyzing their response time (T_res_) and recovery time (T_rec_). The former one is defined as the time taken by Ru-n@PS to finish emission quenching when surrounding atmosphere is switched from pure N_2_ to pure O_2_. The latter one is defined as the time taken by Ru-n@PS to finish emission recovery when surrounding atmosphere is switched from pure O_2_ to pure N_2_. These values are listed in Table 2. It is observed that the response time values of Ru-1@PS and Ru-2@PS samples are two times longer than those of Ru-3@PS samples. This result suggests that the linearly aligned two emissive/sensitive structure indeed brings negative effect to sensing procedure owing to the steric hindrance factor. On the other hand, the response time values of all Ru-n@PS samples are similar to each other and much longer than their response time values. This is because the recovery procedure is controlled by N_2_ diffusion procedure and thus has a correlation only with the microstructure of PS host (Miller and Karpishin, 1999).

## 4 Conclusion

As a conclusion, this work focused on the synthesis and performance comparison of binuclear and mononuclear Ru(II) complexes. Their single crystals were analyzed and theoretically discussed with DFT method. Ru-n complexes all adopted a traditional octahedral coordination sphere at each Ru(II) center. Their onset electronic transition was a mixture of MLCT and LLCT. The contribution ratio of MLCT was higher in binuclear Ru-n complexes, compared with that in mononuclear Ru-n complex (Ru-3). Ru-n complexes were immobilized into PS nanofibers with ES method. Ru-n–doped fibers exhibited improved photophysical performance, compared with pure Ru-n complexes, including improved emission quantum yield, prolonged emission decay dynamics, and emission redshift. Ru-n@PS emission was quenchable by O_2_, showing the highest sensitivity of 6.94 and good photostability. It was found that the sensitivity values of binuclear Ru-n@PS samples (∼6.9) were higher than those of mononuclear Ru-n@PS samples (∼3.3), owing to the relatively high MLCT contribution ratio in emissive center and, consequently, the long emission decay dynamics, which allowed more sensing collision chances with O_2_ molecules. On the other hand, the response time values of binuclear Ru-n@PS samples (15 s) are longer than those of mononuclear Ru-n@PS samples (6 s). The linearly aligned two emissive/sensitive structure indeed brought negative effect to sensing procedure, owing to the steric hindrance factor. The recovery time values of Ru-n@PS samples were similar to each other, with slim correlation with dopant structure. This was because the recovery procedure was controlled by N_2_ diffusion procedure and thus had a correlation only with the microstructure of PS host. The highlight of this work is the realization of linear working curves, which were rarely reported by the previous work. On the other hand, these sensitivity values are still lower than those of oxygen sensing systems based on Pt(II) complexes (∼100). The short excited lifetime of Ru(II)-based probes should be responsible for their underdeveloped sensitivity values. A possible solution is to increase their emission lifetime.

## Data Availability

The datasets presented in this study can be found in online repositories. The names of the repository/repositories and accession number(s) can be found below: (CSD/ICSD, CCDC 1915376, and CCDC 869272, http://www.ccdc.cam.ac.uk/).
