# Supplementary material for: Comparison Between Binuclear and Mononuclear Ru(II) Complexes: Synthesis, Structure, Photophysics, and Oxygen Sensing Performance
Source: Front Chem. 2021 Nov 24;9:785309. doi: 10.3389/fchem.2021.785309 (PMC8651539; doi:10.3389/fchem.2021.785309)
Supplement: Supplementary file 1 [file Table1.DOCX]

Supporting Information

Figure S1. Emission decay dynamics of Ru-1@PS(12%) under pure N_2_ (0.21 μs) and pure O_2_ (0.07 μs) conditions. Monoexponential decay pattern is preserved.


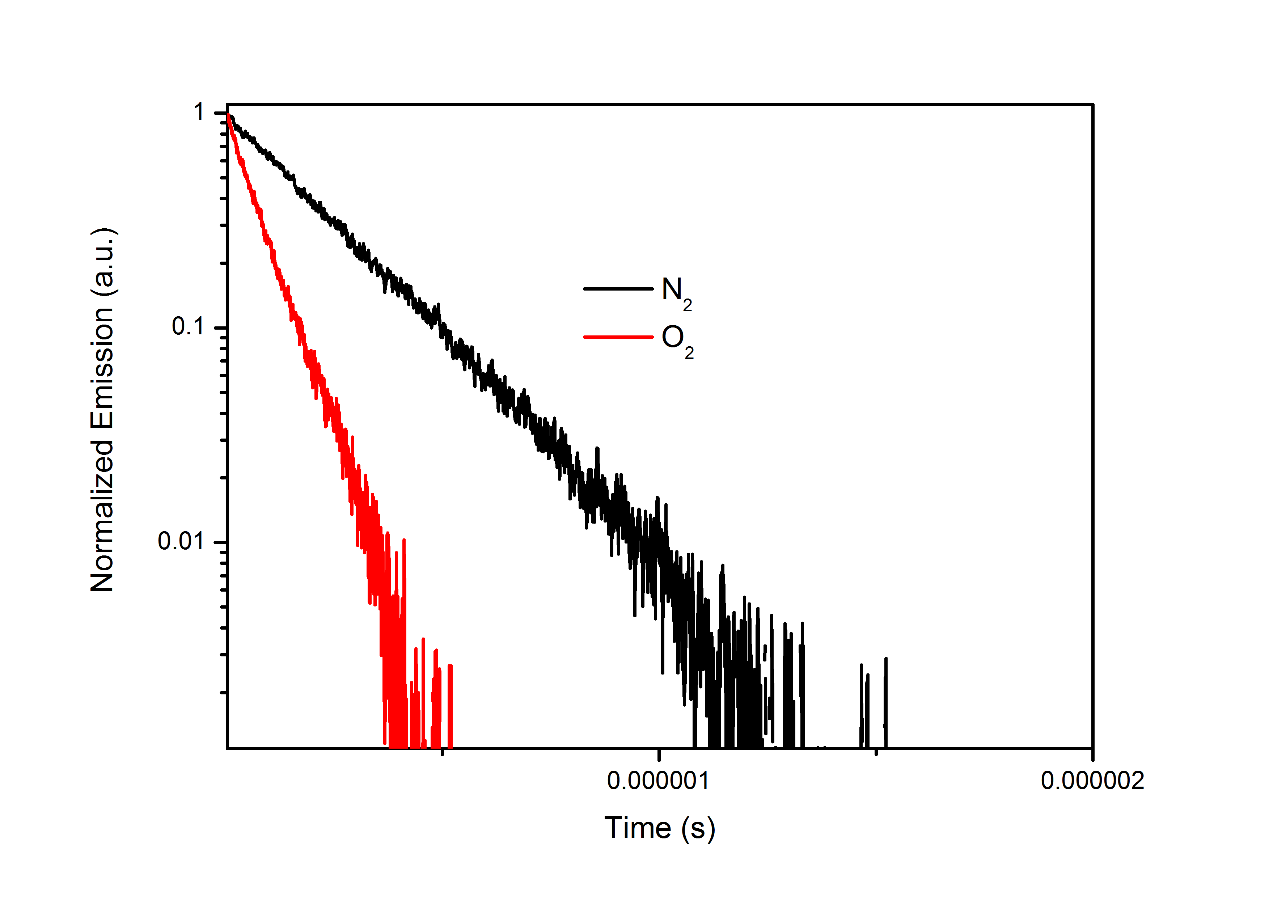


Table S1. Key structural parameters of Ru-1 and Ru-3 single crystals (from CCDC 869272 and 1915376).

| Ru-1 (nuclear #1) | | | | Ru-1 (nuclear #2) | | | |
| --- | --- | --- | --- | --- | --- | --- | --- |
| bond length | (Å) | bond angle | (^o^) | bond length | (Å) | bond angle | (^o^) |
| Ru1-N2 | 2.048 | N2-Ru1-N10 | 78.36 | Ru2-N6 | 2.048 | N6-Ru2-N13 | 79.69 |
| Ru1-N3 | 2.053 | N3-Ru1-N4 | 79.43 | Ru2-N13 | 2.064 | N14-Ru2-N15 | 80.46 |
| Ru1-N4 | 2.045 | N8-Ru1-N11 | 78.57 | Ru2-N14 | 2.066 | N15-Ru2-N16 | 81.46 |
| Ru1-N8 | 2.047 | N2-Ru1-N3 | 86.94 | Ru2-N15 | 2.028 | N6-Ru2-N15 | 95.18 |
| Ru1-N10 | 2.060 | N2-Ru1-N11 | 98.15 | Ru2-N16 | 2.085 | N6-Ru2-N16 | 94.68 |
| Ru1-N11 | 2.061 | N2-Ru1-N4 | 94.45 | Ru2-N17 | 2.040 | N6-Ru2-N16 | 90.29 |
| Ru-3 (mononulcear) | | | |  |  |  |  |
| Ru1-N2 | 2.066 | N2-Ru1-N3 | 79.10 |  |  |  |  |
| Ru1-N3 | 2.073 | N4-Ru1-N8 | 78.35 |  |  |  |  |
| Ru1-N5 | 2.069 | N5-Ru1-N6 | 79.18 |  |  |  |  |
| Ru1-N6 | 2.051 | N2-Ru1-N4 | 89.22 |  |  |  |  |
| Ru1-N4 | 2.060 | N2-Ru1-N5 | 96.10 |  |  |  |  |
| Ru1-N8 | 2.047 | N2-Ru1-N8 | 96.82 |  |  |  |  |

Table S2. Practical sensing performance of Ru-1@PS(12%).

| [O_2_] (%) | detected (%) | error |
| --- | --- | --- |
| 5 | 5.2 | +4% |
| 15 | 15.3 | +2% |
| 35 | 35.9 | +3% |
| 55 | 56.1 | +2% |
| 75 | 76.5 | +2% |
| 95 | 96.7 | +2% |

Detailed geometric prarameters of Ru-1.

Ru1 Ru 0.37377(2) 0.16857(4) 0.08888(2)

Ru2 Ru -0.24542(3) 0.29165(5) 0.17322(3)

N1 N 0.1509(2) -0.1244(4) 0.0257(2)

N2 N 0.3822(2) 0.1215(4) 0.1678(2)

N3 N 0.2941(2) 0.2064(4) 0.0869(2)

N4 N 0.3340(2) 0.0362(4) 0.0552(2)

N5 N -0.1176(2) -0.0795(5) 0.0746(2)

C1 C 0.2468(3) -0.0537(5) 0.0351(2)

C2 C 0.2574(3) 0.1266(5) 0.0709(2)

N6 N -0.2456(2) 0.1475(4) 0.1387(2)

N7 N 0.0191(2) -0.0764(5) 0.0540(2)

N8 N 0.3717(2) 0.2214(4) 0.0122(2)

N9 N 0.1162(2) 0.0200(5) 0.0581(2)

N10 N 0.4081(2) 0.2986(4) 0.1320(2)

N11 N 0.4505(2) 0.1256(4) 0.0820(2)

N12 N -0.0504(2) 0.0420(5) 0.0930(2)

C3 C 0.2022(3) 0.1344(5) 0.0721(2)

C4 C -0.1365(3) 0.0074(6) 0.0959(3)

C5 C -0.1902(3) 0.0246(5) 0.1059(2)

C6 C 0.1837(3) 0.2278(5) 0.0888(3)

H1 H 0.1465 0.2347 0.0900

C7 C 0.1901(3) -0.0469(6) 0.0383(2)

N13 N -0.1680(3) 0.2905(5) 0.1599(3)

C8 C 0.1080(3) -0.0783(6) 0.0386(3)

C9 C -0.2630(5) 0.3824(6) 0.0604(3)

H2 H -0.2243 0.3765 0.0672

C10 C -0.0957(3) 0.0821(6) 0.1073(3)

C11 C -0.2330(3) -0.0471(5) 0.0940(3)

H3 H -0.2289 -0.1134 0.0790

N14 N -0.2371(2) 0.4335(5) 0.2129(2)

C12 C 0.2187(3) 0.3084(6) 0.1032(3)

H4 H 0.2061 0.3732 0.1134

C13 C 0.1706(3) 0.0407(6) 0.0566(2)

C14 C 0.0544(3) -0.1302(6) 0.0342(3)

N15 N -0.2109(3) 0.2434(5) 0.2507(2)

C15 C 0.2791(2) 0.0338(5) 0.0524(2)

C16 C -0.2923(7) 0.4235(8) 0.0085(5)

H5 H -0.2752 0.4485 -0.0184

C17 C -0.3953(5) 0.2353(8) 0.2261(6)

H6 H -0.4078 0.2066 0.2553

C18 C 0.2749(3) 0.2953(5) 0.1030(3)

H7 H 0.2996 0.3515 0.1146

C19 C 0.4100(3) 0.1699(6) 0.2637(3)

H8 H 0.4248 0.2202 0.2911

C20 C 0.5515(3) 0.0811(6) 0.0636(4)

H9 H 0.5865 0.0674 0.0576

C21 C -0.2868(3) 0.0786(6) 0.1272(3)

H10 H -0.3206 0.0967 0.1347

C22 C 0.4028(3) 0.1931(5) 0.2066(3)

C23 C 0.4178(3) 0.2925(5) 0.1861(3)

C24 C 0.0428(3) -0.2298(6) 0.0120(3)

H11 H 0.0687 -0.2660 -0.0025

C25 C 0.5138(3) 0.1311(6) 0.0258(4)

H12 H 0.5218 0.1518 -0.0073

C26 C 0.4627(3) 0.1533(5) 0.0344(3)

C27 C 0.4172(3) 0.2034(5) -0.0061(3)

C28 C 0.3246(3) -0.1366(5) 0.0188(3)

H13 H 0.3411 -0.1955 0.0068

C29 C -0.0298(3) -0.1214(6) 0.0514(3)

C30 C 0.3562(3) -0.0489(5) 0.0371(3)

H14 H 0.3938 -0.0483 0.0369

C31 C -0.0661(3) -0.0528(6) 0.0736(3)

C32 C -0.1975(2) 0.1211(5) 0.1273(3)

N16 N -0.3259(3) 0.2811(5) 0.1799(3)

C33 C 0.2715(3) -0.1391(5) 0.0179(2)

H15 H 0.2505 -0.1997 0.0054

C34 C 0.4215(3) 0.3854(5) 0.1101(3)

H16 H 0.4142 0.3904 0.0715

C35 C 0.4463(3) 0.4700(6) 0.1422(4)

H17 H 0.4562 0.5308 0.1258

C36 C -0.1033(3) 0.1835(6) 0.1283(3)

C37 C -0.2819(3) -0.0176(6) 0.1050(3)

H18 H -0.3123 -0.0640 0.0972

C38 C 0.3276(3) 0.2643(5) -0.0232(3)

H19 H 0.2955 0.2766 -0.0111

C39 C 0.4431(4) 0.3745(6) 0.2208(4)

H20 H 0.4510 0.3674 0.2593

C40 C 0.5399(3) 0.0484(6) 0.1123(3)

H21 H 0.5663 0.0114 0.1394

C41 C 0.3951(4) 0.0751(6) 0.2771(3)

H22 H 0.4005 0.0569 0.3144

C42 C 0.4880(3) 0.0723(5) 0.1192(3)

H23 H 0.4790 0.0498 0.1516

C43 C -0.1548(3) 0.1982(6) 0.1383(3)

C44 C -0.4298(7) 0.2842(12) 0.1774(9)

H24 H -0.4666 0.2951 0.1791

C45 C -0.0436(3) -0.2189(7) 0.0312(3)

H25 H -0.0782 -0.2486 0.0306

C46 C 0.3741(4) 0.2706(7) -0.0926(4)

H26 H 0.3751 0.2879 -0.1287

C47 C 0.3728(4) 0.0068(6) 0.2386(3)

H27 H 0.3608 -0.0588 0.2485

C48 C -0.0056(3) -0.2730(7) 0.0116(4)

H28 H -0.0138 -0.3414 -0.0023

C49 C -0.4201(7) 0.3163(12) 0.1315(8)

H29 H -0.4475 0.3356 0.0996

C50 C 0.3667(3) 0.0297(5) 0.1843(3)

H30 H 0.3511 -0.0211 0.1576

N17 N -0.2846(4) 0.3524(5) 0.0990(3)

C51 C 0.4557(4) 0.4628(6) 0.1983(3)

H31 H 0.4711 0.5200 0.2208

C52 C -0.2170(4) 0.4282(8) 0.2669(4)

C53 C -0.1994(4) 0.3207(9) 0.2878(4)

C54 C 0.4177(4) 0.2275(7) -0.0591(4)

H32 H 0.4495 0.2131 -0.0714

C55 C -0.2299(4) 0.6114(9) 0.2745(4)

H33 H -0.2291 0.6719 0.2963

C56 C 0.3268(4) 0.2910(7) -0.0758(4)

H34 H 0.2953 0.3219 -0.0996

C57 C -0.2525(4) 0.5251(6) 0.1895(3)

H35 H -0.2671 0.5277 0.1510

C58 C -0.3418(6) 0.3537(9) 0.0909(5)

C59 C -0.1298(4) 0.3659(7) 0.1697(4)

H36 H -0.1382 0.4299 0.1845

C60 C -0.1652(6) 0.1982(11) 0.3586(5)

H37 H -0.1507 0.1822 0.3962

C61 C -0.2131(5) 0.5187(9) 0.2974(4)

H38 H -0.1981 0.5151 0.3359

C62 C -0.1725(6) 0.1235(11) 0.3207(5)

H39 H -0.1623 0.0536 0.3307

C63 C -0.2483(4) 0.6158(8) 0.2187(4)

H40 H -0.2580 0.6805 0.2006

C64 C -0.3643(4) 0.3178(8) 0.1367(6)

C65 C -0.1945(4) 0.1488(8) 0.2682(4)

H41 H -0.1987 0.0950 0.2418

C66 C -0.0653(3) 0.2623(8) 0.1399(4)

H42 H -0.0297 0.2525 0.1345

C67 C -0.3399(5) 0.2377(7) 0.2229(5)

H43 H -0.3121 0.2081 0.2516

C68 C -0.1790(6) 0.2991(12) 0.3419(5)

H44 H -0.1743 0.3535 0.3681

C69 C -0.0790(4) 0.3551(8) 0.1593(5)

H45 H -0.0538 0.4116 0.1655

C70 C -0.3503(6) 0.4224(10) 0.0021(6)

H46 H -0.3723 0.4484 -0.0316

C71 C -0.3775(9) 0.3891(13) 0.0379(6)

H47 H -0.4165 0.3883 0.0299

Cl1 Cl 0.30751(8) 0.41437(17) 0.25929(9)

O1 O 0.3138(5) 0.4945(10) 0.2337(9)

O2 O 0.3073(3) 0.3319(6) 0.2288(4)

O3 O 0.3498(3) 0.4146(12) 0.3033(4)

O4 O 0.2548(3) 0.4254(9) 0.2679(4)

Cl2 Cl 0.54583(11) 0.28214(18) 0.36066(10)

O5 O 0.5358(3) 0.2063(5) 0.3190(3)

O6 O 0.5691(6) 0.3595(8) 0.3431(5)

O7 O 0.4914(6) 0.3267(9) 0.3571(6)

O8 O 0.5633(5) 0.2464(11) 0.4133(4)

Detailed geometric parameters of Ru-3.

Ru1 Ru 0.63488(3) 0.35705(4) 0.33510(2)

N1 N 0.4437(3) 0.3970(4) 0.44241(11)

H1 H 0.400933 0.409847 0.437450

N2 N 0.6584(3) 0.3453(4) 0.38204(11)

N3 N 0.5396(3) 0.3771(4) 0.34760(11)

N4 N 0.6616(3) 0.5191(4) 0.33813(11)

N5 N 0.6136(3) 0.1936(4) 0.32749(11)

N6 N 0.6035(3) 0.3567(4) 0.28879(11)

N7 N 0.3770(3) 0.4006(4) 0.49362(11)

N8 N 0.7327(3) 0.3520(4) 0.32627(12)

C1 C 0.5416(3) 0.3763(4) 0.37911(13)

C2 C 0.6049(4) 0.3555(4) 0.39709(13)

C3 C 0.4816(4) 0.3916(5) 0.39144(14)

N9 N 0.5450(3) 0.3620(4) 0.47165(13)

C4 C 0.4898(4) 0.3871(5) 0.42392(14)

C5 C 0.6205(4) 0.6006(5) 0.34436(13)

H2 H 0.576202 0.585103 0.347148

C6 C 0.4217(4) 0.4107(5) 0.37210(15)

H3 H 0.381870 0.422821 0.380084

C7 C 0.7236(4) 0.5435(5) 0.33297(14)

C8 C 0.5515(4) 0.3646(5) 0.44153(14)

C9 C 0.4442(4) 0.3844(5) 0.49789(14)

C10 C 0.6127(4) 0.3478(5) 0.42924(14)

C11 C 0.4204(3) 0.4118(5) 0.34122(15)

H4 H 0.380064 0.424642 0.328028

C12 C 0.5775(4) 0.0596(5) 0.28970(14)

H5 H 0.562624 0.041680 0.269376

C13 C 0.5677(4) 0.2464(6) 0.24445(14)

H6 H 0.556722 0.178253 0.235948

C14 C 0.4804(3) 0.3935(5) 0.32998(14)

H7 H 0.479117 0.392777 0.308928

C15 C 0.6438(4) 0.7084(5) 0.34668(14)

H8 H 0.615353 0.763362 0.351678

C16 C 0.5869(3) 0.2579(5) 0.27614(12)

C17 C 0.5917(3) 0.1669(5) 0.29752(13)

C18 C 0.3840(4) 0.3805(5) 0.54874(15)

C19 C 0.6202(4) 0.1163(5) 0.34856(15)

H9 H 0.634173 0.135671 0.368841

C20 C 0.6004(4) 0.4438(5) 0.27033(14)

H10 H 0.610488 0.511776 0.279119

C21 C 0.7199(4) 0.3233(6) 0.39871(16)

H11 H 0.756682 0.313804 0.388358

C22 C 0.6773(4) 0.3264(5) 0.44522(14)

H12 H 0.683565 0.320498 0.466377

C23 C 0.7315(4) 0.3140(6) 0.43020(16)

C24 C 0.5828(4) 0.4379(6) 0.23879(16)

H13 H 0.582629 0.499474 0.226600

C25 C 0.3465(4) 0.3994(5) 0.51885(15)

C26 C 0.4855(4) 0.3681(5) 0.52687(15)

H14 H 0.532313 0.359122 0.528516

C27 C 0.4546(4) 0.3663(5) 0.55170(16)

H15 H 0.480139 0.355588 0.570908

C28 C 0.7643(4) 0.4486(6) 0.32503(16)

C29 C 0.5848(4) -0.0208(5) 0.31133(16)

H16 H 0.575077 -0.092901 0.305956

C30 C 0.7060(4) 0.7327(6) 0.34180(16)

H17 H 0.721123 0.804399 0.343179

C31 C 0.4795(4) 0.3823(5) 0.47118(15)

C32 C 0.2760(5) 0.4150(7) 0.51546(18)

H18 H 0.250991 0.427161 0.496219

C33 C 0.5654(4) 0.3366(6) 0.22624(14)

H19 H 0.552087 0.329817 0.205267

C34 C 0.6071(4) 0.0083(5) 0.34164(15)

H20 H 0.613140 -0.044353 0.356903

C35 C 0.7490(4) 0.6493(6) 0.33453(17)

H21 H 0.792787 0.664194 0.330883

C36 C 0.7657(4) 0.2613(6) 0.31967(18)

H22 H 0.743997 0.194472 0.320155

C37 C 0.2444(5) 0.4122(8) 0.5403(2)

H23 H 0.197680 0.423558 0.537996

C38 C 0.8278(4) 0.4560(7) 0.3174(2)

H24 H 0.848616 0.523685 0.317287

C39 C 0.2811(5) 0.3924(7) 0.56966(19)

H25 H 0.258267 0.389757 0.586406

C40 C 0.3484(5) 0.3776(6) 0.57358(16)

H26 H 0.371916 0.365015 0.593086

C41 C 0.8305(5) 0.2639(7) 0.3122(2)

H27 H 0.852831 0.199678 0.308803

C42 C 0.8618(4) 0.3639(8) 0.3099(2)

H28 H 0.904030 0.368963 0.303490

Ru2 Ru 0.21179(3) 0.15906(4) 0.35790(2)

N10 N 0.5103(3) 0.1045(4) 0.44463(12)

H29 H 0.545408 0.090772 0.436155

N11 N 0.2227(3) 0.3204(4) 0.34888(13)

N12 N 0.3159(3) 0.1409(4) 0.36166(12)

N13 N 0.2400(3) 0.1698(4) 0.40494(13)

N14 N 0.1100(3) 0.1616(5) 0.35979(14)

N15 N 0.1938(3) -0.0044(4) 0.36260(14)

C43 C 0.3491(4) 0.1375(5) 0.39177(15)

N16 N 0.6275(3) 0.0961(5) 0.48531(13)

N17 N 0.1902(3) 0.1597(5) 0.31123(14)

N18 N 0.4474(4) 0.1359(4) 0.48116(14)

C44 C 0.4561(4) 0.1014(5) 0.37485(15)

H30 H 0.502183 0.084347 0.378853

C45 C 0.5716(4) 0.1134(5) 0.49674(15)

C46 C 0.4204(4) 0.1190(5) 0.39876(15)

C47 C 0.4060(4) 0.1372(5) 0.45321(15)

C48 C 0.4455(4) 0.1186(5) 0.43072(16)

C49 C 0.3077(4) 0.1552(5) 0.41525(16)

C50 C 0.6314(5) 0.1262(5) 0.54772(16)

H31 H 0.632300 0.136059 0.568567

C51 C 0.4242(4) 0.1089(5) 0.34562(15)

H32 H 0.448735 0.101272 0.329541

C52 C 0.3534(4) 0.1284(5) 0.33969(15)

H33 H 0.332119 0.132608 0.319489

C53 C 0.6877(4) 0.0940(6) 0.50452(16)

C54 C 0.3360(4) 0.1568(5) 0.44600(16)

C55 C 0.5081(4) 0.1166(5) 0.47495(17)

C56 C 0.2114(4) 0.3485(6) 0.31881(18)

C57 C 0.2236(5) 0.1913(7) 0.45717(19)

C58 C 0.2909(4) 0.1746(5) 0.46717(17)

H34 H 0.307276 0.174932 0.487990

C59 C 0.2404(4) 0.3996(5) 0.36962(17)

H35 H 0.246814 0.381292 0.390213

C60 C 0.1983(5) 0.1874(6) 0.42579(18)

H36 H 0.152021 0.197190 0.419216

C61 C 0.2396(4) -0.0835(5) 0.36123(17)

H37 H 0.283673 -0.065515 0.358655

C62 C 0.5708(5) 0.1279(5) 0.52838(17)

H38 H 0.530042 0.138258 0.535788

C63 C 0.1289(5) -0.0291(6) 0.3652(2)

C64 C 0.0694(4) 0.2494(7) 0.3586(2)

H39 H 0.088258 0.317068 0.355722

C65 C 0.1913(4) 0.2596(6) 0.29726(16)

C66 C 0.2494(4) 0.5068(5) 0.3617(2)

H40 H 0.262204 0.559546 0.376561

C67 C 0.7584(6) 0.1053(7) 0.5547(2)

H41 H 0.762252 0.116877 0.575663

C68 C 0.6927(5) 0.1095(6) 0.53619(17)

C69 C 0.0811(4) 0.0622(6) 0.3649(2)

C70 C 0.2218(5) -0.1919(6) 0.3636(2)

H42 H 0.253957 -0.246139 0.362567

C71 C 0.1561(5) 0.0803(8) 0.26175(19)

H43 H 0.144911 0.017913 0.250160

C72 C 0.1727(4) 0.0744(6) 0.29278(19)

H44 H 0.171847 0.006162 0.301783

C73 C 0.7455(5) 0.0749(8) 0.4926(2)

H45 H 0.742255 0.064149 0.471645

C74 C 0.2188(4) 0.4554(6) 0.30976(19)

H46 H 0.210129 0.473330 0.289140

C75 C 0.1737(4) 0.2723(7) 0.26595(18)

H47 H 0.173834 0.341428 0.257420

C76 C 0.1562(5) 0.1848(9) 0.2475(2)

H48 H 0.144829 0.192699 0.226492

C77 C 0.0145(5) 0.0547(9) 0.3680(3)

H49 H -0.004355 -0.012825 0.371217

C78 C 0.0023(5) 0.2455(8) 0.3615(3)

H50 H -0.024100 0.308201 0.360139

C79 C 0.8123(6) 0.0857(10) 0.5429(3)

H51 H 0.854287 0.080939 0.555410

C80 C 0.2385(5) 0.5333(6) 0.3306(2)

H52 H 0.244935 0.604337 0.324353

C81 C 0.1572(6) -0.2190(7) 0.3675(3)

H53 H 0.145954 -0.291694 0.369676

C82 C 0.8083(5) 0.0711(9) 0.5104(2)

H54 H 0.847297 0.059357 0.501745

C83 C -0.0248(6) 0.1460(10) 0.3665(4)

H55 H -0.070301 0.140624 0.368771

C84 C 0.1080(6) -0.1391(7) 0.3681(3)

H56 H 0.063612 -0.156483 0.370314

P1 P 0.37126(10) 0.26046(14) 0.25422(4)

F1 F 0.4273(2) 0.2040(3) 0.27922(8)

F2 F 0.4077(2) 0.2121(3) 0.22759(9)

F3 F 0.4186(2) 0.3678(3) 0.25572(8)

F4 F 0.3352(2) 0.3082(4) 0.28120(9)

F5 F 0.3250(2) 0.1538(3) 0.25333(11)

F6 F 0.3153(3) 0.3168(4) 0.22955(10)

P2 P 0.56621(11) 0.25047(15) 0.63754(4)

F7 F 0.5108(3) 0.1763(4) 0.64961(11)

F8 F 0.5097(3) 0.3381(4) 0.62410(11)

F9 F 0.5738(4) 0.3183(4) 0.66847(12)

F10 F 0.6202(3) 0.3251(5) 0.62554(18)

F11 F 0.6208(3) 0.1692(5) 0.65350(19)

F12 F 0.5537(4) 0.1874(5) 0.60634(13)

P3 P 0.8053(2) 0.0223(3) 0.40000(10)

F13 F 0.7434(5) 0.0727(7) 0.4102(2)

F14 F 0.8728(6) -0.0274(9) 0.3909(4)

F15 F 0.8180(9) 0.1268(15) 0.3862(4)

F16 F 0.7766(10) -0.0934(13) 0.3975(5)

F17 F 0.8334(16) 0.028(3) 0.4300(7)

F18 F 0.7766(12) 0.020(3) 0.3665(4)

P4 P 1.0273(3) 0.5513(5) 0.32011(19)

F19 F 0.9809(5) 0.5116(9) 0.3440(3)

F20 F 0.9644(5) 0.5682(12) 0.2963(4)

F21 F 1.0904(6) 0.5271(9) 0.3444(4)

F22 F 1.0303(6) 0.4290(9) 0.3084(3)

F23 F 1.0217(10) 0.6731(12) 0.3284(4)

F24 F 1.0734(6) 0.5841(16) 0.2972(5)

O1 O 0.6152(4) 0.0150(5) 0.41665(16)

H57 H 0.654306 0.037849 0.417998

C85 C 0.6165(6) -0.0951(7) 0.4256(2)

H58 H 0.572420 -0.126721 0.419349

H59 H 0.628383 -0.099957 0.447353

H60 H 0.649564 -0.133576 0.416068

O2 O 0.2959(4) 0.4873(5) 0.43842(19)

H61 H 0.314596 0.450462 0.452745

C86 C 0.3151(6) 0.5988(8) 0.4433(2)

H62 H 0.275218 0.643738 0.440111

H63 H 0.344857 0.619423 0.429184

H64 H 0.338253 0.607999 0.463799

O3 O 0.1566(9) 0.4574(13) 0.4346(4)
